# Structural Insights Into m6A-Erasers: A Step Toward Understanding Molecule Specificity and Potential Antiviral Targeting

**DOI:** 10.3389/fcell.2020.587108

**Published:** 2021-01-12

**Authors:** Mahmoud Bayoumi, Muhammad Munir

**Affiliations:** ^1^Division of Biomedical and Life Sciences, Lancaster University, Lancaster, United Kingdom; ^2^Virology Department, Faculty of Veterinary Medicine, Cairo University, Giza, Egypt

**Keywords:** ALKBH5, antiviral, demethylation, epigenetics, evolution, FTO, structural insights, m6A

## Abstract

The cellular RNA can acquire a variety of chemical modifications during the cell cycle, and compelling pieces of evidence highlight the importance of these modifications in determining the metabolism of RNA and, subsequently, cell physiology. Among myriads of modifications, methylation at the N6-position of adenosine (m^6^A) is the most important and abundant internal modification in the messenger RNA. The m^6^A marks are installed by methyltransferase complex proteins (writers) in the majority of eukaryotes and dynamically reversed by demethylases such as FTO and ALKBH5 (erasers). The incorporated m^6^A marks on the RNA transcripts are recognized by m6A-binding proteins collectively called readers. Recent epigenetic studies have unequivocally highlighted the association of m^6^A demethylases with a range of biomedical aspects, including human diseases, cancers, and metabolic disorders. Moreover, the mechanisms of demethylation by m^6^A erasers represent a new frontier in the future basic research on RNA biology. In this review, we focused on recent advances describing various physiological, pathological, and viral regulatory roles of m^6^A erasers. Additionally, we aim to analyze structural insights into well-known m^6^A-demethylases in assessing their substrate binding-specificity, efficiency, and selectivity. Knowledge on cellular and viral RNA metabolism will shed light on m^6^A-specific recognition by demethylases and will provide foundations for the future development of efficacious therapeutic agents to various cancerous conditions and open new avenues for the development of antivirals.

## Introduction

Epitranscriptome is an emerging area of biology that collectively describes over 100 chemical modifications to various forms of RNAs, including messenger RNA (mRNA), transfer RNA (tRNA), ribosomal RNA, and long non-coding RNAs (lncRNAs). These chemical modifications display an extensive landscape that regulates multiple biological processes (Roundtree et al., 2017). RNA can accept one or more chemical modifications to different bases, including cytosine (m^5^C) (Motorin et al., 2009), adenosine (m^1^A) (Li X. et al., 2017; Safra et al., 2017), pseudouridine (Carlile et al., 2014), and inosine (Levanon et al., 2004). However, methylation at the N6 position of adenosine (m^6^A) is considered the most prominent modification (Dominissini et al., 2012; Boccaletto et al., 2018). In addition to cellular RNA, the m6A marks are also incorporated into the viral RNA (Krug et al., 1976; Kane and Beemon, 1985; Narayan et al., 1987; Tirumuru et al., 2016; Courtney et al., 2017; Kennedy et al., 2017), hence highlighting unexplored aspects of host–pathogen interactions.

During the physiological regulatory processes, the methylation process is embarked on by the m^6^A methyltransferase complex. Conversely, to reverse the m6A marks, the RNA demethylases are required to alleviate the effects of various installed chemical modifications and/or dynamically reverse RNA changes to perform a specified function in cell life cycles (Han et al., 2010). Various mammalian alkylated DNA repair protein (AlkB) homologs share the same basic structure to nine publicly known AlkB protein members (Sundheim et al., 2008; Yang et al., 2008; Aik et al., 2012; Wang et al., 2014). The prototype AlkB gene/protein was firstly identified in *Escherichia coli* strains in the 80s (Kataoka et al., 1983); however, the detailed functions of AlkB proteins in repairing the damage arise from alkylation were described in the 2000s. The bacterial AlkB protein has a broad range of specificity to various nucleobases (Falnes et al., 2002; Delaney and Essigmann, 2004; Delaney et al., 2005; Alemu et al., 2016). Unlike the multifunctional prokaryotic AlkB, the higher-order eukaryotic AlkB homologs, such as ALKBH1-8 and the FTO, have only limited functions with higher substrate specificity for either epigenetic modifications and/or nucleic acids repair function (Falnes et al., 2002).

Human AlkB Homolog-1 (hALKBH1) protein was first documented to repair 3-methylcytosine (3mC) in both DNA and RNA (Westbye et al., 2008). The hALKBH1 was identified to mediate additional lyase activity of DNA at abasic sites in Fe^2+^- or 2-oxoglutarate-independent manner (Müller et al., 2010). Moreover, it has been reported that ALKBH1 regulates post-transcriptional gene expression through promoting methylation reversal of N1-methyladenosine (m^1^A) in both cytoplasmic and mitochondrial tRNAs (Liu et al., 2016; Kawarada et al., 2017). Furthermore, mammalian ALKBH1 demethylates m^5^C derivative intermediates on the tRNAs as well in various cellular compartments (Kawarada et al., 2017). More recently, it was confirmed that ALKBH1 could also demethylate N6-methyladenine (m^6^A) on DNA, suggesting dual important epigenomic regulatory roles in DNA and epitranscriptomic roles on various forms of RNAs (Tian et al., 2020; Zhang et al., 2020). Although ALKBH-2 and−3 promote both m^1^A and 3-methylcytidine (m^3^C) demethylation, ALKBH2 efficiently repairs both methylated single-stranded DNA (ssDNA) and double-stranded DNA (dsDNA), whereas ALKBH3 preferentially demethylates single-stranded nucleic acids (Monsen et al., 2010). Recently, ALKBH3 was found to post-transcriptionally regulate protein expression through the demethylation of m^1^A on specific cellular transcripts (Woo and Chambers, 2019). Besides this role, ALKBH3 demethylates specific tRNA modifications, including m^1^A and m^3^C, which ultimately promotes cancer progression (Chen Z. et al., 2019). Of all ALKBHs described so far, ALKBH-4 and−7 were found to demethylate preferentially proteins rather than nucleic acids (Li et al., 2013; Wang et al., 2014). Importantly, the widely studied eukaryotic ALKB homologs proteins including ALKBH5 and FTO were found to specifically demethylate m^6^A, which is the most prevalent internal chemical modification on RNA for epigenetic control of cell life cycles (Jia et al., 2011; Zheng et al., 2013; Feng et al., 2014; Xu et al., 2014). Moreover, ALKBH8 was reported to mediate 5-methoxycarbonylmethyluridin repair through hydroxylation of tRNA (Fu et al., 2010). The detailed function of ALKBH6 has not yet been identified (Hu et al., 2019).

Herein, we aim to provide a comprehensive review of the recent progress made to uncover the structural features of the m^6^A demethylases compared with the rest of the AlkB protein members. Additionally, we aim to draw comparative features between ALKBH5 and FTO for their binding specificity, efficiency, and selectivity along with providing the recent updates of the various regulatory aspects of m^6^A erasers and the promising inhibitors to further guide the development of efficacious therapeutics to target cancers, metabolic disorders, and viruses.

### Enzymatic Biochemistry of m^6^A Demethylases

The identification of different nucleobases that had been exposed to oxidative demethylation is deemed essential for understanding the intracellular biological and metabolic functions of the m^6^A-containing substrates. Confined mostly to the nucleus, ALKBH5 utilizes the m^6^A-containing ssRNA as the major substrate for demethylation *via* α-ketoglutaric-dependent oxidase activity (Aik et al., 2014; Feng et al., 2014). The ALKBH5 has also been reported to target the dimethylated adenosine (m${}_{2}^{6}$A) in the ribosomal RNA. The m${}_{2}^{6}$A is a non-canonical base present in ribosomal RNA as a normal component of the small subunit of the ribosome that assists in the common translation machinery (Ensfelder et al., 2018).

The Schofield group was the first to predict the earliest substrate for FTO, the 3-methylthymine (3mT), *via* bioinformatic analysis (Gerken et al., 2007). Consistent with the functional analysis that exhibited human and murine expressed FTOs repair the 3mT preferentially in ssDNA over dsDNA and favorably demethylate the 3-methyluracil (3mU) in ssRNA over ssDNA (Jia et al., 2008).

The m^6^A was confirmed to be catalyzed by FTO both *in vivo* and *in vitro* (Jia et al., 2011; Wei et al., 2018; Zhang X. et al., 2019). Furthermore, the +1 position to 5'cap in the polyadenylated RNA was confirmed to be di-methylated at N6 and 2'-O-position (m^6^A_m_) as a major substrate for FTO that regulates the 5' mRNA integrity, stability, and resistance to decapping enzyme (e.g., DCP2) (Mauer et al., 2017). Intriguingly, the latter study claimed that the m^6^A_m_ is the sole physiological substrate for demethylation than m^6^A by FTO. This finding diametrically opposes most compelling evidence stating the relevant substrates of FTO (Jia et al., 2011; Fu et al., 2013; Wei et al., 2018; Zhang X. et al., 2019). It is worth noting that the hepatitis C virus (HCV), an ssRNA virus that belongs to the *Flaviviridae* family, was confirmed to harbor m^6^A marks throughout the entire viral RNA and respond to demethylation activity of FTO despite lacking the 5'cap (Gokhale et al., 2016). Additionally, recent investigations have identified that lacking the m^6^A_m_ methyltransferase does not affect the cell growth kinetics and vital cellular processes (Akichika et al., 2019). In contrast, detrimental cellular alterations were observed in FTO knockdown cells (Zhao et al., 2014a; Li Z. et al., 2017). More recently, Sendinc et al. have illustrated that phosphorylated C-terminal domain (CTD) interacting factor-1 is an m^6^A_m_ methyltransferase and m^6^A_m_ is an evolutionarily conserved modification to the capped mRNAs. However, no crosstalk between the m^6^A and m^6^A_m_ was detected in the whole transcriptome mapping. Additionally, m^6^A_m_ promotes gene regulation mainly through mediating protein translation but not the transcription or mRNA stability (Sendinc et al., 2019). Interestingly, another report emphasizes the non-significant effect of phosphorylated CTD interacting factor-1 on protein translation (Boulias et al., 2019).

**Figure 1:**
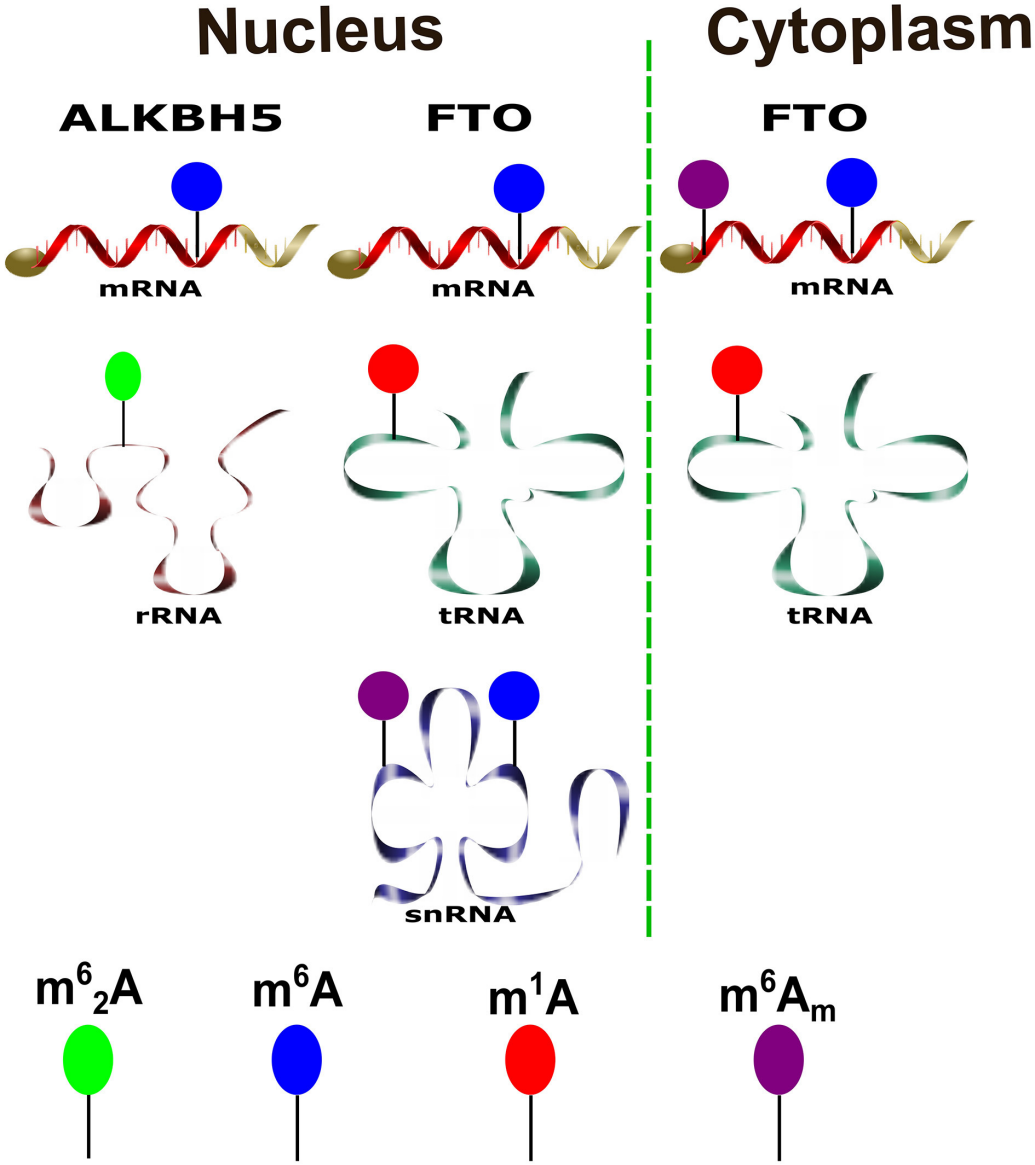
Various physiological substrates of the m^6^A demethylases. A schematic diagram shows substrates of ALKBH5 and FTO and their distribution in both cytoplasm and nucleus to the different forms of RNAs inside the cell.

Systematically, Wei et al. have investigated the differential FTO substrate preference along with their location in various cell lines. The FTO preferentially mediates methylation reversal of the internal m^6^A in both the cytoplasm and nucleus on the polyadenylated RNAs. The percent of demethylation differs according to the investigated cell line. In contrast, FTO-mediated m^6^A_m_-polyA RNA demethylation was confined to the cytoplasm (Wei et al., 2018). Moreover, the biochemical studies have identified additional RNA substrates to FTO in the various forms of RNA, including N1-methyladenosine (m^1^A) in tRNA located in both nucleus and cytoplasm. It is important to note that m^1^A-demethylated tRNAs have prominent action on translation efficiency (Liu et al., 2016; Wei et al., 2018). Moreover, it was confirmed that both m^6^A and cap m^6^A_m_ in small nuclear RNAs all found to be substrates for FTO that might control gene expression (Wei et al., 2018). Various physiological substrates for m6A-demethylases are summarized in Figure 1.

### Structural Insights of the m^6^A Demethylases Determine Their Substrate Specificities

Our group has performed a recent comprehensive analysis of the m^6^A demethylases among various orders of animals, especially the avian species (Bayoumi et al., 2020). The study revealed multiple evolutionary changes when compared with *Homo sapiens*. We have revealed that m^6^A-erasers shared the lowest identity percent among the m^6^A-related machinery. However, the overall demethylases' structures were maintained through synonymous structural mutations (Bayoumi et al., 2020). The entire human AlkB-homolog-5 (hALKBH5) protein comprises a polypeptide chain of 394 amino acids (Zheng et al., 2013; Huang and Yin, 2018). Owing to technical challenges that have been experienced in the processing of the apo ALKBH5 enzyme in both *in vitro* enzymatic and crystallographic investigations, including those harboring different ligands, ALKBH5_66−292_ truncated fragment was active for functional and structural studies as well (Aik et al., 2014; Feng et al., 2014). The 65 N-terminus- and 103 C-terminus-residues were not essential for ALKBH5 core oxidative demethylation activity to targeted substrates. However, the C-terminus multiple serine residues were supposed to mediate phosphorylation (Aik et al., 2014).

From the earlier mentioned eukaryotic ALKBH protein family, all shared basic scaffold structure dubbed as jelly-roll [or double-stranded β-helix (DSβH)] fold, which is composed of conserved eight anti-parallel β-sheets in almost all species (Jia et al., 2011; Aik et al., 2012, 2014; Bayoumi et al., 2020). Besides the basic jelly-roll fold, additional secondary structures (nucleotide recognition motifs) were characterized in most ALKBH protein family members. It can be concluded that the basic scaffold has no substrate specificity function, whereas the secondary structures carry some level of specificity. Notably, no secondary structures were identified in both the ALKBH-4 and−7. Therefore, no oxidative methylation activity was detected toward nucleic acids and was only confined to the protein substrates (Li et al., 2013; Wang et al., 2014). From the substrate specificities mentioned earlier, it seems that adenosine (A) is the sole nucleobase to ALKBH5 in ssRNA (Aik et al., 2014; Feng et al., 2014; Xu et al., 2014).

**Figure 2:**
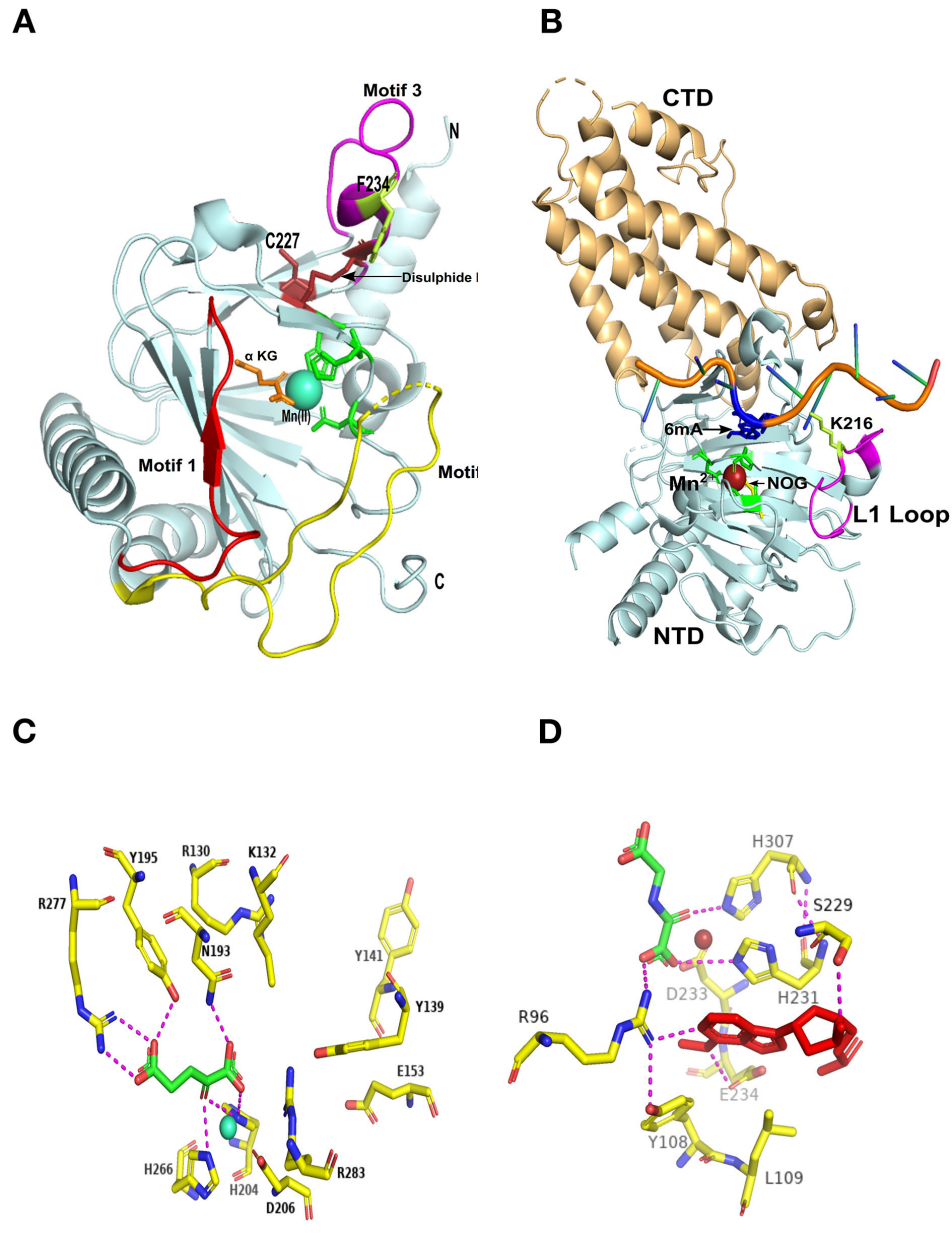
Structural comparison between ALKBH5 and FTO. **(A)** Overall three-dimensional structure of ALKBH5 (PDB ID: 4NRO); the overall jelly-roll fold is colored in polycyan; the secondary recognition motifs-1,−2, and−3 are colored red, yellow, and magenta, respectively; disulfide bond depicted by an arrow; conserved HxD..H motifs are represented by green residues; motif-3 F234 is represented by limon residue; C227 is represented by firebrick residue; alpha-ketoglutaric acid (α-KG) is represented by orange residue; manganese atom is represented by cyan circle. N: N-terminus, C: C-terminus. **(B)** Overall three-dimensional structure of FTO (PDB ID: 5ZMD); C-terminal domain (CTD) is colored polycyan; N-terminal domain (NTD) is colored light orange; unique loop (L1) is colored magenta; conserved HxD..H motifs are represented by green residues; K216 is represented by limon residue; 2-oxoglutarate analog (NOG) is represented by yellow residue; 6-methyladenine substrate (6mA) is represented by blue color; manganese atom is represented by firebrick circle. **(C)** ALKBH5 active site residues (PDB ID: 4NRO) (yellow carbon residues identified by their numbers); alpha-ketoglutaric acid (α-KG) is represented by green carbon residue, attached by active site residues by magenta covalent bonds; manganese atom is represented by cyan circle. **(D)** FTO active site residues (PDB ID: 5ZMD) (yellow carbon residues identified by their numbers); 2-oxoglutarate analog (NOG) is represented by green carbon residue; and the 6-methyladenine nucleobase (6 mA) is represented by red color attached to the active site residues by magenta covalent bonds; manganese atom is represented by firebrick circle.

Several groups have worked independently to illustrate the crystallographic analysis of the human ALKBH5 harboring various substrates and inhibitors (Aik et al., 2014; Feng et al., 2014; Xu et al., 2014). All of these groups have identified three unique amino acid motifs (Figure 2A). The motif 1, the position of this motif in relation to the active catalytic site, provides a widening surface compared with FTO and ALKBH2 (Feng et al., 2014), which proposes that the ALKBH5 can tolerate bulker three-dimensional structure substrates for targeted oxidative demethylation (Aik et al., 2014). Additionally, motif 2 was identified as a long motif that provides flexibility compared with other AlkB proteins (Feng et al., 2014). Notably, motif 3 has been confirmed to impede the double-stranded nucleic acid substrates that confirms ALKBH5 selectivity to an only single-stranded nucleic acid (Feng et al., 2014).

In addition to the conserved active site coordinated residues (HXD…H, motif), the basic residues adjacent to active sites (in motif 1) were also found to be crucial for enzymatic activity, including K132 (Figure 2C). This was identified to interact with m^6^A and can also accept additional post-translational modifications (e.g., acetylation) that helps the enzymatic oxidative demethylation (Choudhary et al., 2009; Aik et al., 2014). The mutant K132A was identified to severely impair the ALKBH5 activity (Feng et al., 2014). Furthermore, ALKBH5 R130 residue, which was located in the unique motif 1 (Figures 2A,C), was supposed to interact directly with the single-stranded phosphate backbone (Aik et al., 2014). This interaction was confirmed by complete abrogation of the catalytic activity through targeted-mutational studies (Feng et al., 2014).

Likewise, within the long motif 2, unique amino acids were also identified to interact with m^6^A single-stranded substrate that confers substrate specificity, including Q146, K147, and R148 residues. Additionally, this is characterized by reduced demethylation activities (40%) upon their targeted mutations (Sundheim et al., 2006; Yang et al., 2008; Han et al., 2010; Feng et al., 2014). Most importantly, ALKBH5 motif 3 (Figure 2A) was implicated as the main secondary structure in the outer wall of DSβH; however, this motif is also present in other AlkB members (McDonough et al., 2010). The motif specifically flips in a way to impede with double-stranded substrates displaying steric hindrance by covalent disulfide bonding. This bond is conserved among various species of ALKBH5 between C230 and C267 or alternatively connects the C227 thiol group through redox shuffle mechanism generating C227-C267 linkage (Figure 2A). This mechanistically confers single stranded substrates selectivity. Moreover, F234 residue has been found to interact and direct the m^6^A-containing substrate toward the active catalytic site. The residues mentioned earlier were detected to be evolutionary conserved when tested by site-directed mutagenesis to ALKBH5 that specifically ensure strand specificity and secondary structure confirmation. Furthermore, the electrostatic map around the active catalytic site is important for the substrate binding. Mutational analysis found that more basic surfaces mainly to the active sites and the grooves made by the protrusion of the long motif 2 was pivotal for binding with the negative phosphate backbone form single-stranded substrates for optimal oxidative demethylation (Aik et al., 2014; Feng et al., 2014). Collectively, structural insights and the unique motifs and residues could be exploited to provide a better understanding of the substrate- and nucleotide-specificity for upcoming biomedical basic researches and development of ALKBH5 selective inhibitors.

Similar to the AlkB member family, FTO has the conserved jelly-roll motif (DSβH) harboring the active catalytic site in its N-terminal domain (NTD) (1-326). However, a novel fold designated as CTD (from 327 to 498 aa) has been structurally determined and is supposed to strengthen the NTD (Figure 2B). The publicly available crystal structure of FTO lacking the first 31 amino acids still retains the full enzymatic functionality indicating the active site buried in NTD and stabilized by CTD (Han et al., 2010). Likewise, the selectivity of ALKBH5 against the unmethylated strand of double-stranded nucleic acid, FTO, was also identified to harbor an evolutionary stretch of amino acid residues named long loop 1 (L1; residues from 210 to 223) (Figure 2B). We and others have confirmed that the L1 loop is identified in *H. sapiens* and avian species and unidentified in the rest of AlkB members; this unique loop selectively blocks dsDNA/RNA to serve as a physiological substrate for FTO (Han et al., 2010; Feng et al., 2014; Zhang X. et al., 2019; Bayoumi et al., 2020).

Concerning the putative physiological substrates, it seems that the FTO outperforms the ALKBH5 in the number of physiological substrates to demethylate their methylated nucleobases. FTO promotes oxidative methylation to m^6^A and m^6^A_m_ in both mRNA and snRNA, and m^1^A in tRNA. Furthermore, FTO demethylates 6mA, 3mT, and m3U (Han et al., 2010; Jia et al., 2011; Wei et al., 2018; Zhang X. et al., 2019). This array of substrates toward an AlkB member emphasizes the distinctiveness of the catalytic activity to accommodate various nucleobases. Besides the selectivity to hinder the double-stranded nucleic acids, the L1 loop has been investigated through biochemical and structural analysis to contribute to nucleobases recognition and stabilization of the single-stranded substrate in the FTO active site (Figure 2B; Zhang X. et al., 2019). Comprehensively, the L1 loop represented by K216 from one side and the short loop (residues 86–88) represented by K88 form hydrogen bonds with the phosphate backbones of the nucleotides adjacent to the methylated nucleobase. These lysine residues act as a pincer-like structure in twisting and accommodating the target nucleobase in the catalytic pocket (Figure 2B). Moreover, inside the catalytic pocket, the methylated base is stabilized by the hydrophobic interaction with the surrounding residues: I85, L109, Y108, V228, S229, W230, and H231. In contrast, the N6-methyl group specifically is stabilized in the pocket by the hydrophobic interaction with Y106, L203, and R322 residues (Figure 2D). Importantly, the methylated purine ring interacts with R96 and E234 predominantly by hydrogen bonding, whereas the ribose ring interacts mostly with A229 (Zhang X. et al., 2019). Therefore, the targeted mutations to these hydrogen bonding interacting residues abrogated the demethylation activity (Zhang X. et al., 2019). The same findings were also observed in other ALKBH homologs to residues corresponding to the R96. The site-directed mutation of M61 residue in AlkB and Q112 in ALKBH2 diminished their enzymatic functions (Han et al., 2010), suggesting highly conserved demethylation among various AlkB family members. Albeit, we reported the lowest identity percent of the avian FTO compared with the *H. sapiens*. A higher degree of conservation to the residues surrounding the methylated base in both *H. sapiens* and avian species was noticed, indicating a highly conserved catalytic mechanism even in various organisms exhibiting evolutionary changes. Moreover, we have found that the pincer-like structure in avian species suggests a higher binding affinity with more stabilizing property compared with *H. sapiens* (Bayoumi et al., 2020).

Considering the challenge of the similarity that could affect m^6^A antibody mismatching with m^6^A_m_, high-throughput sequencing can differentiate them throughout the transcriptome (Linder et al., 2015). Compared with the m^6^A distribution across the mRNA, the m^6^A_m_ was documented to be located less frequently (Molinie et al., 2016). At least a 10-fold higher m^6^A level than that of the cap m^6^A_m_ in mRNA was confirmed (Wei et al., 2018; Zhang X. et al., 2019). Because the same nucleobase (i.e., adenosine) between m^6^A and m^6^A_m_ were noticed, FTO superimposition studies exhibited the same oxidative demethylation activity in the same RNA sequence, with no significant effect to the ribose sugar on the enzymatic activity. However, the N6-methyl adenine group was confirmed to surpass other nucleobases to accommodate the active site of FTO, and 3meT was the lowest. Unequivocally, all mentioned substrates contain all the pivotal structural determinants for FTO physiological substrates to accommodate the active site. Moreover, the wide pincer-like structure formed by the unique loop one in FTO can accommodate higher numbers of substrates rather than ALKBH5 with bulkers secondary and tertiary structures such as the cap, stem-loop, and hairpin structures (Zhang X. et al., 2019).

Zou and co-workers have adopted detailed biophysical and biochemical analyses to determine the specificity of m^6^A demethylases in the nucleotide perspectives. They confirmed that both ALKBH5 and FTO do not exhibit strict sequence requirements for their substrates as other m^6^A-recognizing proteins; writers and readers do. Moreover, m6A demethylases can recognize and differentiate m^6^A marks in the highly similar nucleotide sequences, even having the same consensus motif, with superiority to the FTO. Notably, erasers can induce different outcomes in different RNA sequences, with different secondary structure conformation (duplex to hairpin transition), concluding that m^6^A itself is considered as a conformational marker (Zou et al., 2016).

### Biological Functions of the m^6^A Demethylases

The m^6^A demethylases (ALKBH5 and FTO) modulate various aspects of cell life cycles that can diverge from the regulation of normal metabolic and differentiation functions, which aggravates numerous pathological conditions. In the past few decades, multiple tumor processes were documented across the literature with poor underlying molecular genetic justifications. After that, the field of epigenetics has become a relevant topic to provide possible explanations for several human diseases (Pinello et al., 2018; Chen X. Y. et al., 2019; Huang et al., 2020; Melstrom and Chen, 2020; Zhao et al., 2020).

#### Pathological Regulatory Aspects of m6A Demethylases

Epigenetically, the m^6^A demethylases dictate the fate of various cancerous conditions. In the thoracic cancers, Forristal et al. have investigated the effects of reduced O_2_ tension (5%) on the upregulation of certain hypoxia-inducible factors (HIFs) in comparison with human embryonic stem cell control maintained in normoxic condition (20%). They have noticed the translocation of HIFs to the nucleus to reduce O_2_ tension condition (Forristal et al., 2010). The translocated HIF-1α protein transcriptionally activates multiple targets as a cellular response to the hypoxia, chief among them was the human ALKBH5 gene (Thalhammer et al., 2011). The ALKBH5 plays an important role in controlling breast cancer progression through the HIF-ALKBH5-dependent pathway. ALKBH5 demethylates m^6^A marks from NANOG, a master pluripotency factor; the oxidative demethylation activity of the ALKBH5 increases the NANOG transcript and protein expression that enriches breast cancer stem cells in the reduced oxygen tumor microenvironment promoting cancer progression (Zhang et al., 2016a). After that, Zhang et al. have also reported that knockdown of ALKBH5 from breast cancer cells could suppress breast-to-lung metastasis in mice model (Zhang et al., 2016b). Furthermore, FTO contributes to breast cancer development. It has been found that FTO overexpression was associated with a higher incidence of human breast cancer. FTO m^6^A-mediated demethylation of 3'- untranslated region BNIP3 transcript, which is a proapoptotic protein belonging to the Bcl-2 tumor suppressor family, promoting its degradation *via* YTHDF2 independent pathways and specific upregulation of BNIP3 retards breast cancer proliferation and metastasis (Niu et al., 2019). Collectively, it seems that thoracic cancer progression is controlled negatively by specific mRNA methylation reversal (Deng et al., 2018a,c; Mauer and Jaffrey, 2018; Pinello et al., 2018; Rajecka et al., 2019; Melstrom and Chen, 2020). More recently, the elevation of the ALKBH5 level was also confirmed to be involved in lung adenocarcinoma proliferation and invasion under intermittent hypoxia conditions. ALKBH5 demethylates Forkhead box M1 (FOXM1), which is one of the main tumor inducers. Upon m^6^A demethylation, the FOXM1 transcript provides stabilization of the expressed protein (Chao et al., 2020). The ALKBH5 has also been demonstrated to regulate the tumorigenic progression of oral squamous cell carcinoma that antagonizes the utilized chemotherapeutics for the intervention of proliferation and metastasis (Shriwas et al., 2020). The ALKBH5-dependent demethylation of FOXM1 and NANOG transcripts (main regulators of cancer stem cells) promotes chemoresistance of platinum-based drugs through negative regulation of human DEAD-box RNA helicase (DDX3), which are primarily involved in the innate immunity, multiple cell signal processes, and numerous aspects of RNA metabolism (Shriwas et al., 2020).

Despite ALKBH5 has been identified to contribute significantly to physiological osteogenesis (Yu et al., 2020), ALKBH5 mediates osteosarcoma (OS) tumorigenesis *via* demethylation of plasmacytoma variant translocation one, a tumorigenic lncRNA. Mechanistically, ALKBH5 removes the m^6^A marks, increases the stability of mRNA, and enhances the expression of plasmacytoma variant translocation one through inhibiting its YTHDF2 binding, resulting in increased OS cell proliferation rates both *in vitro* and *in vivo* (Int et al., 2020). Similar to the OS tumorigenesis, ALKBH5 possesses a negative regulatory impact in gastric cancer (GC) *via* acting on another lncRNA named nuclear paraspeckle assembly transcript one that results in enhancement of EZH2 expression (a component of the polycomb repressive complex) and ultimately affects the invasion and metastasis in GC tissues (Zhang J. et al., 2019; Zhu et al., 2020). The same fate was identified in FTO overexpression in GC cancer tissues compared with adjacent non-tumorous tissue (Xu et al., 2017; Zhang C. et al., 2019). Taken together, it seems that m^6^A erasers demethylate both mRNA and lncRNA to promote carcinogenesis and have a negative oncogenic signature in multiple cancers.

Likewise, m^6^A demethylases modulate sex-specific tumors. Marked expression of ALKBH5 has been detected in ovarian cancer, which mediates the EGFR-PIK3CA-AKT-mTOR-signaling pathway, a key regulatory pathway in autophagy-induced stress response and nutrient deprivation. Additionally, ALKBH5 enhances the stability of the BCL-2 transcript (which increased in the epithelial ovarian cancer as well) and enhances the interaction between BCL-2 and Beclin1 that inhibit the autophagy from the other side, suggesting that the ALKBH5 controls tumor progression and autophagy flux *via* BCL-2 demethylation (Zhu et al., 2019). In contrast, in males, the ALKBH5 was found to control testicular germ cell tumors type II (Nettersheim et al., 2019).

Not only that soft tissue tumors are controlled epigenetically, but FTO has also been incriminated in the progression of the solid tumor, including melanoma. Two mechanisms were proposed, through single-nucleotide polymorphisms outside of intron one (body mass index-related region), as rs16953002, the variant of intron 8 of FTO that has been reported to be associated with a high risk of melanoma (Iles et al., 2013; Deng et al., 2018b). Additionally, the FTO was identified as a pro-tumorigenic factor in melanoma. The FTO negatively regulates the response to anti-programmed death 1, an immunotherapeutic agent, through the action of melanoma-intrinsic genes including PD-1, C-X-CR-4, and SOX10; those are the major potential gene targets for demethylation by FTO (Yang S. et al., 2019; Melstrom and Chen, 2020; Zhao et al., 2020).

Great focus has been dedicated to deciphering the oncogenic role of FTO in hematopoietic disorders. These include acute myeloid leukemia through promoting leukemogenesis *via* FTO-mediated m^6^A demethylation of core transcripts as ASB2 and RARA mRNAs promoting decreased stability of the target transcripts (Li Z. et al., 2017; Huang et al., 2019; Weng et al., 2019; Zhao et al., 2020). Additionally, ALKBH5 was found to be linked with the devastating malignant brain tumor glioblastoma through the ALKBH5-FOXM1-mediated pathway; in this milieu, ALKBH5 enhances glioblastoma tumorigenesis (Dixit et al., 2017; Zhang et al., 2017; Malacrida et al., 2020).

Unlike the fate of the cancers mentioned earlier, the m^6^A demethylases alleviate the outcome of additional biological processes. ALKBH5 expression was noticed to be downregulated in pancreatic tumors. ALKBH5 targets a lncRNA named KCNK15-AS1 *via* direct demethylation and is associated with inhibition of the pancreatic cancer metastasis, which might serve as a potential therapeutic target for pancreatic cancer patients (He et al., 2018). More recently, mechanistic investigations have documented another ALKBH5-mediated inhibition of the most common form of pancreatic cancers, the pancreatic ductal adenocarcinoma, through the ALKBH5 dependent-Wnt inhibitory factor one pathway (Tang et al., 2020). To conclude, ALKBH5 carries suppressive effects on certain tumors to provide mounting evidence to be an excellent new prognostic marker for pancreatic cancers (Cho et al., 2018; Melstrom and Chen, 2020).

Similar findings were noticed with bladder cancer repression through the action of the ALKBH5 and METTL3 in a reciprocal manner on integrin alpha-6 transcript, which enhances various cellular motility and signaling events. The ALKBH5 inhibits the translation of integrin alpha-6 in the m^6^A-dependent pathway and decreases bladder cancer adhesion, migration, and invasion (Jin et al., 2019). Moreover, colon cancer was suppressed upon overexpression of the ALKBH5 in both cell invasion *in vitro* and metastasis *in vivo* (Yang P. et al., 2019). Thus, ambitious therapeutic candidates have also been proposed in head and neck squamous cell carcinoma *via* overexpression of ALKBH5 and FTO (PilŽys et al., 2019). To conclude, various actions of m^6^A demethylases were noticed to either suppress or enhance cancer development and progression through direct oxidative demethylation on either specific mRNAs or lncRNAs. Additionally, accumulating evidence suggests using m^6^A demethylases or their gene targets for either prognostic and diagnostic markers for specific tumors as indicated earlier, and improving specific inhibitors for future use could open a new frontier in alleviating multiple cancerous conditions.

#### Metabolic and Physiological Regulatory Roles of m6A Demethylases

It is well-documented that m^6^A-containing mRNA regulates various biological processes, including autophagy, which is an evolutionarily conserved degradation pathway in the cell. A critical association between the autophagy from one side and METTL3-ALKBH5 interplay from the other side has been found to control hypoxia/reoxygenation-treated cardiomyocytes (*in vitro* and in an animal model) in which the ALKBH5 acted as a positive regulator in the autophagy *via* regulating m^6^A level on the transcription factor EB mRNA and its subsequent protein expression. Transcription factor EB is the main regulator of autophagy-related genes and ultimately regulates the fate of ischemic heart diseases (Song et al., 2019).

Additionally, the obesity problem in humans has been linked to the FTO. Albeit, obesity is concomitant to various inherited and behavioral determinants that further predisposes to other chronic diseases; the FTO is also incriminated in adipogenesis. FTO single-nucleotide polymorphisms, which are mostly located in intron-1, were found to be linked with obesity in humans (Zhao et al., 2014b). There are multiple proposed mechanistic regulatory roles of FTO in the development and progression of obesity (Gulati et al., 2013). In contrast, others suggested that the FTO gene is under the control of nearby associated genes, chief among them IRX3 to be the main regulator in obesity (Smemo et al., 2014). However, the obesity–FTO associations are reviewed well elsewhere (Zhou et al., 2017; Deng et al., 2018b; Mauer and Jaffrey, 2018).

Vis-à-vis eraser's physiological roles, the ALKBH5 has been found to play a pivotal role in the regulation of the enrichment of the human placenta during pregnancy via the action on trophoblasts that seems to affect the recurrent miscarriage patients. Mechanistic studies have revealed that ALKBH5 mediates the action by affecting the half-life of cysteine-rich angiogenic inducer-61 mRNA that possesses differentiation, migration, and adhesion roles, which are important for normal embryogenesis (Li et al., 2019). Furthermore, FTO was found to be involved in premature ovarian insufficiency-mediated infertility. The reduction of FTO protein expression was concomitant with elevated m^6^A level in ovarian tissue of premature ovarian insufficiency patients (Ding et al., 2018). A similar finding reported in male mice has a deficiency in ALKBH5. Those mice were identified to have increased levels of m^6^A in their transcripts, consequently impaired fertility and apoptosis along with the ultimate negative effect on the meiotic metaphase stage of the spermatocytes (Zheng et al., 2013). Tang et al. (2017) have unveiled the mechanistic insights of ALKBH5-mediated m^6^A's role in male infertility and revealed that ALKBH5 ensured the production of longer 3′-untranslated region transcripts coupled with correct splicing (Tang et al., 2017). Regarding differentiation functions of demethylases, the ALKBH5 regulates multiple metabolic processes as adipogenesis and myogenesis through modulating the early differentiation markers such as CEBPb and myogenin, respectively (Choi et al., 2019). The FTO was also found to play roles in differentiating the neuronal stem cells in adult mice (Cao et al., 2019). The various pathological and physiological regulatory roles of m6A-demethylases are summarized in Table 1.

**Table 1:** Regulatory aspects of m^6^A demethylases.

**m^**6**^A demethylase**	**Regulatory aspect**	**Tissue involved**	**Regulatory Gene(s) & their expression level**	**References**
ALKBH5	Cancer type	Breast cancer	↑NANOG	Zhang et al., 2016a,b
		Glioblastoma	↑FOXM1	Dixit et al., 2017; Zhang et al., 2017; Malacrida et al., 2020
		Lung adenocarcinoma	↑FOXM1	Chao et al., 2020
		Pancreatic cancer	↑KCNK15-AS1 ↑WIF- 1	He et al., 2018 Tang et al., 2020
		Bladder cancer	↓ITGA6	Jin et al., 2019
		Oral squamous cell carcinoma	↑FOXM1 / NANOG	Shriwas et al., 2020
		Osteosarcoma	↑PVT1	Int et al., 2020
		Gastric cancer	↑NEAT1	Zhang J. et al., 2019; Zhu et al., 2020
		Colon cancer		Yang P. et al., 2019
		Ovarian cancer	↑Bcl2	Zhu et al., 2019
		Male germ cell tumor		Nettersheim et al., 2019
	Metabolic disorder	Male infertility	↑Correct spliced/longer transcripts	Tang et al., 2017
		Autophagy (ischemic heart disease)	↑TFEB	Song et al., 2019
	Differentiation	Placenta	↓CYR61	Li et al., 2019
		Adipogenesis	↓CEBPb	Choi et al., 2019
		Myogenesis	↓Myogenin	Choi et al., 2019
FTO	Cancer type	Breast cancer	↓BNIP3	Niu et al., 2019
		Melanoma	↑PD-1 CXCR4 SOX10	Yang S. et al., 2019; Melstrom and Chen, 2020; Zhao et al., 2020
		Acute myeloid leukemia	↓ASB2 and RARA	Li Z. et al., 2017; Huang et al., 2019; Weng et al., 2019; Zhao et al., 2020
		Gastric cancer		Xu et al., 2017; Zhang C. et al., 2019
	Metabolic disorder	Obesity	FTO gene Intron1 IRX3	Zhao et al., 2014b Smemo et al., 2014
		Premature ovarian insufficiency		Ding et al., 2018
	Differentiation	Neuronal stem cells		Cao et al., 2019

#### Viral Regulatory Aspects of m6A Demethylases

Similar to cellular transcripts, viral RNA can accept the decoration by m^6^A to regulate/dictate the viral life cycle and outcome of virus–host interactions (Dang et al., 2019). These include multiple viruses of medical importance as well as oncogenic viruses. The m^6^A-demethylase-mediated modification could control viral replication, pathogenesis, infection, and ultimately tumor formation (Imam et al., 2018; Tan et al., 2018; Tsai et al., 2018; Lang et al., 2019). The hepatitis B virus (HBV) is a DNA tumor-causing virus linked with chronic hepatitis, a high risk of liver cirrhosis, and hepatocellular carcinoma (Shepard et al., 2006). HBV intermediate transcripts have been confirmed to bear m^6^A marks from both hepatic tissues of chronic HBV patients and HBV-expressing cells (Imam et al., 2018). Furthermore, m6A machinery represented by METLL3, METTL14 from one side, and FTO from the other side mediates two major regulatory functions. Firstly, the viral gene expression and secondly the reverse transcription based on the m6A modified site on the epsilon loop of HBV that modulate the fate of HBV in the liver disease pathogenesis and tumor formation (Imam et al., 2018).

Moreover, Kaposi's sarcoma-associated herpesvirus (KSHV) is another salient example of a human oncogenic virus linked with different cancers, including Kaposi's sarcoma and primary effusion lymphoma; KSHV has latent and lytic replication stages in the lifecycle (Ye et al., 2011). Recent advances in epitranscriptome sequencing revealed that m^6^A could modulate the transition between the stages with altered m^6^A methylome, and erasers modulate the lytic gene expression that controls KSHV infection and KSHV-induced oncogenesis. Recent studies have reported that m^6^A modifications play different roles owing to various cell types during lytic replication of KSHV (Ye et al., 2017; Hesser et al., 2018; Tan et al., 2018).

Additionally, Epstein–Barr virus is another example of oncogenic herpes viruses caused by human herpesvirus-4, which is incriminated with 2% of human cancers. Through the interplay of METTL14, YTHDF2, and ALKBH5, Epstein–Barr virus latent protein EBNA3C is responsible for reprogramming the methylome that enhances tumorigenesis via the m^6^A-dependent pathway (Lang et al., 2019). Similarly, the Simian virus 40, a DNA oncogenic virus, and HCV, a major RNA tumor-causing virus, are impacted positively or negatively through the m6A-dependant pathways, respectively (Gokhale et al., 2016; Tsai et al., 2018). However, mechanistic action of FTO or ALKBH5 for tumor formation remains to be identified.

Additionally, the non-oncogenic viruses are m^6^A decorated as well, and the m^6^A demethylases have an intriguing role in different virus life cycles. During virus infection, the ALKBH5 only induces a regulatory role in virus replication and protein expression as reported previously in human immunodeficiency virus-1 (HIV-1) and vesicular stomatitis virus (VSV) (Lichinchi et al., 2016a; Tirumuru et al., 2016; Liu et al., 2019). In contrast, in others, the FTO only modulates viral infection, including HCV (Gokhale et al., 2016) and enterovirus-71 (Hao et al., 2019). However, in the case of the Zika virus and respiratory syncytial virus, both demethylases have regulatory functions (Lichinchi et al., 2016b; Xue et al., 2019). Cumulatively, it is plausible that m^6^A demethylases display various regulatory functions in different cell contexts (even those infected with the same virus model), likely through regulating distinct sets of targets, suggesting more detailed analysis for the near future and for designing the correct specific inhibitor. Additionally, future systematic studies will determine the biological function of each of the m^6^A regulatory genes in various cancer settings and the critical target genes to unveil the underlying molecular mechanisms.

### m^6^A Demethylases' Inhibitors

**Figure 3:**
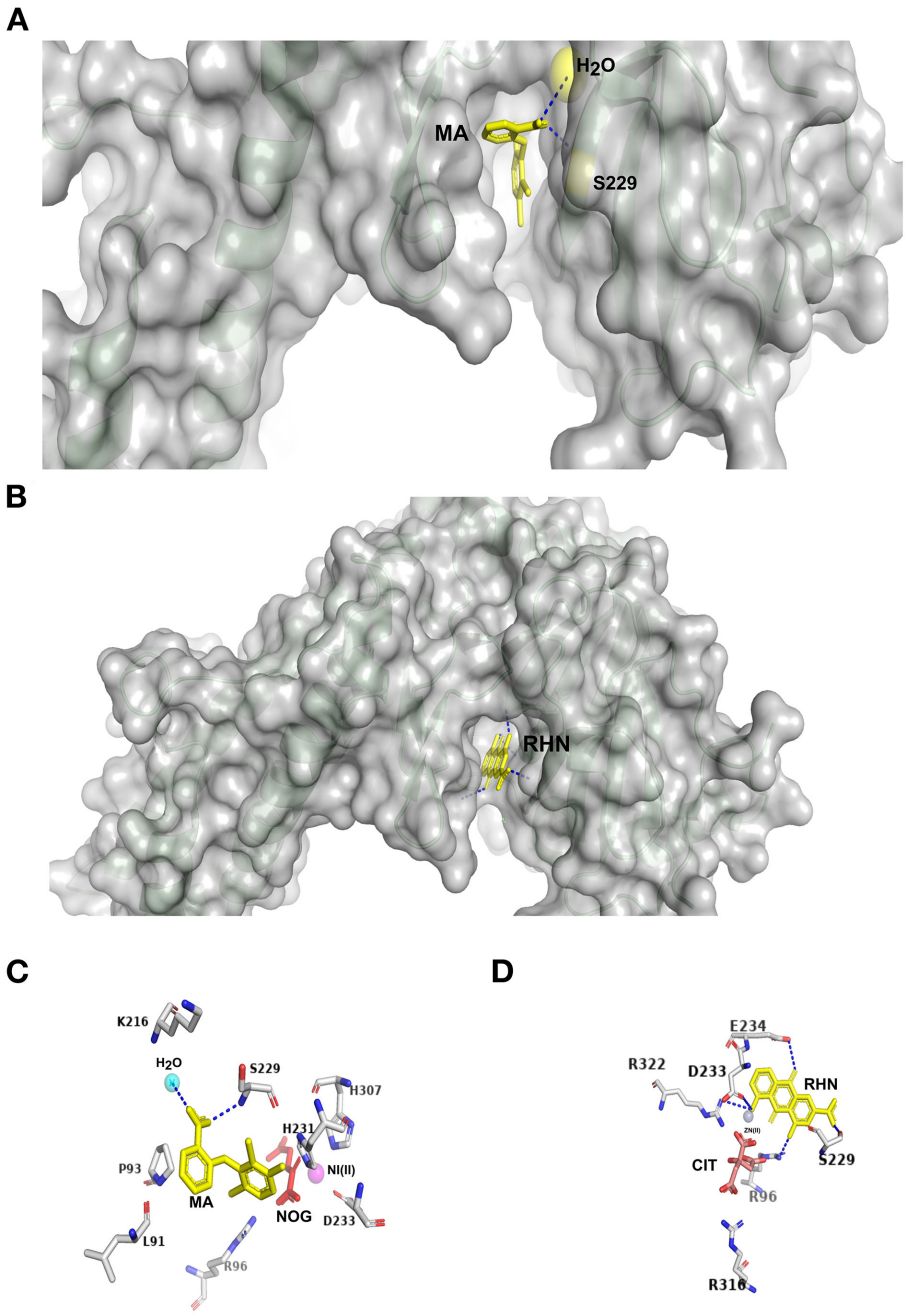
Specific recognition of inhibitors within FTO. **(A)** Meclofenamic acid (MA) binds to the deep pocket on the surface of FTO (PDB ID: 4QKN) (represented by gray semitransparent surface); MA represented by yellow residue binds to both H_2_O yellow circle and S229 yellow residue by blue covalent bonds. **(B)** Rhein RHN binds to the deep pocket on the surface of FTO (PDB ID: 4IE7) (represented by gray semitransparent surface); RHN is represented by yellow residue binds to the active site residues by blue covalent bonds. **(C)** Detailed view of meclofenamic acid (MA) within FTO (PDB ID: 4QKN) active residues (gray carbon residues identified by their numbers); MA is represented by yellow color residue, attached to the active site residues by blue covalent bonds; 2-oxoglutarate analog (NOG) is represented by red carbon residues; nickel (Ni) atom is represented by a pink circle. H_2_O is represented by a pink circle. **(D)** Detailed view of Rhein (RHN) within FTO (PDB ID: 4IE7) active residues (gray carbon residues identified by their numbers); RHN is represented by yellow color residue, attached by active site residues by blue covalent bonds; citrate molecule (CIT) is represented by red residue; zinc (Zn) atom is represented by a dark gray circle.

Unraveling m^6^A demethylases structures along with a better understanding of their physiological and tumorigenic regulatory roles inspired various groups to develop different types of inhibitors to impede the enzymatic activity. Modulating the m^6^A level inside cells is an ambitious target to control various cancerous condition invasion and metastasis as discussed earlier. Therefore, inhibition of the prototype *E. coli* AlkB was the proof-of-concept to this notion using a natural product named quercetin (Chen et al., 2012). Importantly, with the availability of the FTO crystallographic structure (Han et al., 2010), a comprehensive mechanistic study to utilize cell-active, natural products (rhein) was confirmed to reversibly bind to the nucleotide-binding pocket *in vitro* and inside cells with reduced cell toxicity. Structurally, the positively charged active site (R316) of FTO was found to interact with the negatively charged carboxyl group of the rhein to hinder m^6^A repair (Figures 3B,D; Chen et al., 2012). Additional wide arrays of FTO small-inhibitor molecules were suggested to abolish FTO catalytic activity *via* either interacting with the nucleotide-binding and/or 2OG binding sites (Figure 3; Aik et al., 2013).

After that, fluorescence polarization studies with chemical displacement have been utilized to validate the use of meclofenamic acid (MA), an anti-inflammatory drug, to provide temporal intervention of mRNA methylation. The MA competes with the m^6^A-binding site (Figures 3A,C) and inhibits FTO over ALKBH5 (Huang et al., 2015). It is worth noticing that MA was reported to be successfully used for inhibition of FTO demethylation activity in the KSHV lifecycle and has been confirmed to enhance the lytic gene expression in comparable results with FTO loss-of-function experiments (Ye et al., 2017). Despite the potent activity of the rhein and MA, inhibiting other essential cellular enzymes were noticed to shut down their activities (Chen et al., 2012; Flanagan et al., 2012; Li et al., 2016; Huang et al., 2019). Wang et al. have utilized the structural similarity between some fluorescein compounds to MA to selectively inhibit FTO activity and provide additional labeling simultaneously (Wang et al., 2015).

Additionally, MO-I-500, a pharmacologically tested FTO inhibitor, was also reported reducing the survival rate of inflammatory breast cancer cell lines selectively (Singh et al., 2016). Moreover, a robust tool has been recognized depending on the difference in both substrate and nucleotide specificities, which provides compounds that occupy both nucleotide and 2OG binding pockets. This method is named the two-component inhibitor tethering strategy (Toh et al., 2015).

Rational drug design through the scaffold hopping approach was also adopted to identify new candidates for FTO inhibitors. These candidates were tested using docking simulations. Structural analysis of MU06-bounded-FTO revealed interaction of R96 and H231 of FTO catalytic pocket with MU06 inhibitor via H bonding (Padariya and Kalathiya, 2016). Recently, fluorescent RNA aptamers were utilized as a tool for studying FTO inhibitors in a high-throughput screening format (Svensen and Jaffrey, 2016). Additional natural compounds were identified as putative FTO inhibitors such as radicicol (Wang et al., 2018) and clausine E (Wang et al., 2019). Other compounds have additional medicinal advantages, such as the anti-epileptic effect (Zheng et al., 2014) and the anti-leukemic activity of the R-2HG (Su et al., 2018).

More recently, promising FTO inhibitors such as FB23 and FB23-2 were selected and tested in an animal model. It was found to impede FTO in a way mimicking FTO depletion in acute myeloid leukemia cell lines. Structurally, these inhibitors have complementarity with the substrate-binding pocket *via* binding with the critical residues in the active site, including S229, R96, and E234 (Huang et al., 2019; Figures 3C,D).

Although most of the compound, as mentioned earlier, can totally or partially inhibit FTO, the MV1035, an imidazobenzoxazin-5-thione, was initially synthesized as a Na^+^ channel blocker, using structural-based *in silico* screening in the wide-scale proteome. MV1035 was found to interact with ALKBH5 as an off-target molecule. After that, the functional analysis was confirmed to fight the glioblastoma aggressiveness (Malacrida et al., 2020).

## Concluding Remarks: How Viruses Can Provide More Information for A Better Understanding of Epigenetics in The Future

Methylation of viral RNA has recently been considered as a hallmark in virus–host interactions. Viral epitranscriptome allows us to underpin molecular mechanisms of m^6^A modification and its impact on cellular and viral RNAs behaviors. It has been concluded that the activity of the writers and the readers were associated with restraining the viral replication capacity. In contrast, the demethylases exert an opposite effect in virus-infected cells, suggesting an overall negative regulation of viral replication (Brocard et al., 2017).

Furthermore, m^6^A is proposed to negatively regulate interferon (IFN) responses in virus–host interaction. Significant reduction in various dsDNA viruses (including human cytomegalovirus, HCMV) titers was reported in m^6^A-writers and readers knockout (KO) cells, and marked elevation to viral titers were noticed in FTO- or ALKBH5-KO cells along with the fast turnover of IFN transcripts, hence facilitating viral propagation (Winkler et al., 2019). Mechanistically, cellular RNA helicase (DDX46) inhibits the innate immune response through the DDX46-ALKBH5-dependant pathway, leading to the demethylation of IFN transcripts. Demethylation of these mRNAs enforces their retention in the nucleus and inhibits IFN production and consequently enhances viral propagation (Zheng et al., 2017).

However, this is not the case for all viruses investigated so far. Interestingly, m^6^A has a positive regulatory outcome of certain viruses such as HIV-1. The depletion of the METTL3 and/or METTL14 (writers) has been confirmed to inhibit HIV-1 replication, whereas knockdown of the ALKBH5 enhances the replication (Kennedy et al., 2016, 2017; Tirumuru et al., 2016). The same findings were consistent in enterovirus-71, which is another ssRNA virus. In the enterovirus-71 replication model, the viral RNA copy number and protein expression were regulated mainly by the FTO. Intriguingly, the ALKBH5 does not affect the virus lifecycle (Hao et al., 2019). Moreover, the enhanced viral gene expression and replication have also been reported in the influenza A virus (Courtney et al., 2017) and SV-40 (Tsai et al., 2018). However, the m6A demethylases' roles in viral replication have not been investigated in greater detail.

In contrast, the negative impact of m^6^A demethylases was noticed in the HCV (Gokhale et al., 2016), Zika (Lichinchi et al., 2016b), and VSV (Liu et al., 2019). It is important to note that depletion of FTO was concomitant with a reduced infectious virus and HCV RNA release. Interestingly, ALKBH5 does not influence the HCV life cycle (Gokhale et al., 2016). ALKBH5 and FTO enhance the titer and the protein synthesis of the Zika virus, which is another member of the *Flaviviridae* family (Lichinchi et al., 2016b; Tan and Gao, 2018). Notably, it was also confirmed that knockdown of ALKBH5 significantly reduced VSV RNA levels (Liu et al., 2019). Despite intensive studies of epitranscriptome to cellular RNA, the molecular events illustrating virus–cell epitranscriptome interactions are in their infancy, and several fundamental questions need to be answered. Especially, m^6^A demethylases, as the focal point of this review, must understand differences between FTO and ALKBH5 in their pattern of recognition to closely related viral RNA.

Considering the m6A mark as a conformational marker, the sequence variation and secondary and tertiary structures between two viruses, which belong to the same family, could be the cause of preferential target to one demethylase compared with another. However, detailed structural and functional studies warrant further investigation that might reveal aspects in understanding the mechanistic action toward viruses to provide efficacious antivirals in the near future.

Moreover, detailed studies of all m^6^A-related proteins (writers, readers, and erasers) could explain the various outcomes against different viruses. This was not surprising, as loss-of-function studies of m^6^A-demethylases have different outcomes in various cancerous conditions, as discussed earlier. Additionally, certain viruses were found to accept the variant of m^6^A modification (i.e., m^6^A_m_), which can affect the fate of virus replication (Lichinchi et al., 2016b; Tirumuru et al., 2016; Tan et al., 2018). Interestingly, FTO is the unique demethylase that interacts and responds to m^6^A_m_. However, detailed crystallographic analysis of FTO harboring m^6^A and/or m^6^A_m_ could provide more answers in both cellular and viral epitranscriptomic field.

Considering the splicing function regulated by both demethylases (Zheng et al., 2013; Bartosovic et al., 2017), their role in the splicing process of viruses (DNA viruses, HIV, and influenza A virus) warrants further investigation. Besides, the discrepancies in the various reports in cellular and/or viral epitranscriptome might be owing to variation cell type, site of modifications, the utilized technique for sequencing (Tan et al., 2018; Dang et al., 2019). However, the viral epigenetic is at the stage of infancy and warrants exhaustive research in the near future.

Given the roles of m^6^A-demethylases in multiple virus life cycles and tumorigenic capacity shed light on the future potential use of inhibitors to fight a wide range of biological process simultaneously. The proof-of-concept has been provided from the data described in multiple studies. The use of various FTO inhibitors such as broad-spectrum m^6^A inhibitor named 3-deazaadenosine (DAA) *in vitro* and animal model (Kennedy et al., 2016; Courtney et al., 2017) and the specific FTO inhibitor (MA) in KSHV model (Ye et al., 2017) provide valuable insights. These proof of principle studies underline the applicability of m^6^A “demethylases” inhibitors in developing next-generation antiviral and cancer therapies.

## Author Contributions

MM: conceptualization, supervision, writing—review, and editing. MB and MM: formal analysis, investigation, and resources. MB: writing—original draft preparation. All authors contributed to the article and approved the submitted version.

## Conflict of Interest

The authors declare that the research was conducted in the absence of any commercial or financial relationships that could be construed as a potential conflict of interest.

## References

[B1] Aik W. Demetriades M. Hamdan M. K. K. Bagg E. A. L. Yeoh K. K. Lejeune C.. (2013). Structural basis for inhibition of the fat mass and obesity associated protein (FTO). J. Med. Chem. 56, 3680–3688. 10.1021/jm400193d 23547775

[B2] Aik W. Scotti J. S. Choi H. Gong L. Demetriades M. Schofield C. J. (2014). Structure of human RNA N6-methyladenine demethylase ALKBH5 provides insights into its mechanisms of nucleic acid recognition and demethylation. Nucleic Acids Res. 42, 4741–4754. 10.1093/nar/gku085 24489119 PMC3985658

[B3] Aik W. S. McDonough M. A. Thalhammer A. Chowdhury R. Schofield C. J. (2012). Role of the jelly-roll fold in substrate binding by 2-oxoglutarate oxygenases. Curr. Opin. Struct. Biol. 22, 691–700. 10.1016/j.sbi.2012.10.001 23142576

[B4] Akichika S. Hirano S. Shichino Y. Suzuki T. Nishimasu H. Ishitani R.. (2019). Cap-specific terminal N 6 -methylation of RNA by an RNA polymerase II–associated methyltransferase. Science 363, 1–13. 10.1126/science.aav0080 30467178

[B5] Alemu E. A. He C. Klungland A. (2016). ALKBHs-facilitated RNA modifications and de-modifications. DNA Repair. 44, 87–91. 10.1016/j.dnarep.2016.05.026 27237585 PMC5120542

[B6] Bartosovic M. Molares H. C. Gregorova P. Hrossova D. Kudla G. Vanacova S. (2017). N6-methyladenosine demethylase FTO targets pre-mRNAs and regulates alternative splicing and 3′-end processing. Nucleic Acids Res. 45, 11356–11370. 10.1093/nar/gkx778 28977517 PMC5737695

[B7] Bayoumi M. Rohaim M. A. Munir M. (2020). Structural and virus regulatory insights into avian N6-methyladenosine (m6A) machinery. Front. Cell Dev. Biol. 8:543. 10.3389/fcell.2020.00543 32760718 PMC7373739

[B8] Boccaletto P. MacHnicka M. A. Purta E. Pitkowski P. Baginski B. Wirecki T. K.. (2018). MODOMICS: a database of RNA modification pathways. 2017 update. Nucleic Acids Res. 46, 303–307. 10.1093/nar/gkx1030 29106616 PMC5753262

[B9] Boulias K. Toczydłowska-Socha D. Hawley B. R. Liberman N. Takashima K. Zaccara S.. (2019). Identification of the m6Am methyltransferase PCIF1 reveals the location and functions of m6Am in the transcriptome. Mol. Cell 75, 631–643.e8. 10.1016/j.molcel.2019.06.006 31279658 PMC6703822

[B10] Brocard M. Ruggieri A. Locker N. (2017). m6A RNA methylation, a new hallmark in virus-host interactions. J. Gen. Virol. 98, 2207–2214. 10.1099/jgv.0.000910 28869001

[B11] Cao Y. Zhuang Y. Chen J. Xu W. Shou Y. Huang X.. (2019). Dynamic effects of Fto in regulating the proliferation and differentiation of adult neural stem cells of mice. Hum. Mol. Genet. 29, 727–735. 10.1093/hmg/ddz274 31751468

[B12] Carlile T. M. Rojas-Duran M. F. Zinshteyn B. Shin H. Bartoli K. M. Gilbert W. V. (2014). Pseudouridine profiling reveals regulated mRNA pseudouridylation in yeast and human cells. Nature 515, 143–146. 10.1038/nature13802 25192136 PMC4224642

[B13] Chao Y. Shang J. Ji W. (2020). ALKBH5-m6A-FOXM1 signaling axis promotes proliferation and invasion of lung adenocarcinoma cells under intermittent hypoxia. Biochem. Biophys. Res. Commun. 521, 499–506. 10.1016/j.bbrc.2019.10.145 31677788

[B14] Chen B. Ye F. Yu L. Jia G. Huang X. Zhang X. (2012). Development of cell-active N 6 -methyladenosine RNA demethylase FTO inhibitor. J. Am. Chem. Soc. 134, 17963–17971. 10.1021/ja3064149 23045983

[B15] Chen X. Y. Zhang J. Zhu J. S. (2019). The role of m6A RNA methylation in human cancer. Mol. Cancer 18, 1–9. 10.1186/s12943-019-1033-z 31142332 PMC6540575

[B16] Chen Z. Qi M. Shen B. Luo G. Wu Y. Li J.. (2019). Transfer RNA demethylase ALKBH3 promotes cancer progression via induction of tRNA-derived small RNAs. Nucleic Acids Res. 47, 2533–2545. 10.1093/nar/gky1250 30541109 PMC6411830

[B17] Cho S. H. Ha M. Cho Y. H. Ryu J. H. Yang K. Lee K. H. (2018). ALKBH5 gene is a novel biomarker that predicts the prognosis of pancreatic cancer: a retrospective multicohort study. Ann. Hepato-Biliary-Pancreatic Surg. 22:305 10.14701/ahbps.2018.22.4.305 30588520 PMC6295372

[B18] Choi S. Y. Choi M. J. Cho M. Y. Park Y. J. (2019). Alkbh5, a RNA demethylase, is involved in fine-tuning of cell differentiation (FS11-07-19). Curr. Dev. Nutr. 3:7051274 10.1093/cdn/nzz037.FS11-07-19

[B19] Choudhary C. Kumar C. Gnad F. Nielsen M. L. Rehman M. Walther T. C.. (2009). Lysine acetylation targets protein complexes and co-regulates major cellular functions. Science 325, 834–840. 10.1126/science.1175371 19608861

[B20] Courtney D. G. Kennedy E. M. Dumm R. E. Bogerd H. P. Tsai K. Heaton N. S.. (2017). Epitranscriptomic enhancement of influenza A virus gene expression and replication. Cell Host Microbe 22, 377–386.e5. 10.1016/j.chom.2017.08.004 28910636 PMC5615858

[B21] Dang W. Xie Y. Cao P. Xin S. Wang J. Li S.. (2019). N6-Methyladenosine and viral infection. Front. Microbiol. 10:417. 10.3389/fmicb.2019.00417 30891023 PMC6413633

[B22] Delaney J. C. Essigmann J. M. (2004). Mutagenesis, genotoxicity, and repair of 1-methyladenine, 3-alkylcytosines, 1-methylguanine and 3-methylthymine in alkB *Escherichia coli*. Proc. Natl. Acad. Sci. U.S.A. 101, 14051–14056. 10.1073/pnas.0403489101 15381779 PMC521119

[B23] Delaney J. C. Smeester L. Wong C. Frick L. E. Taghizadeh K. Wishnok J. S.. (2005). AlkB reverses etheno DNA lesions caused by lipid oxidation *in vitro* and *in vivo*. Nat. Struct. Mol. Biol. 12, 855–860. 10.1038/nsmb996 16200073

[B24] Deng X. Su R. Feng X. Wei M. Chen J. (2018a). Role of N 6 -methyladenosine modification in cancer. Curr. Opin. Genet. Dev. 48, 1–7. 10.1016/j.gde.2017.10.005 29040886 PMC5869081

[B25] Deng X. Su R. Stanford S. Chen J. (2018b). Critical enzymatic functions of FTO in obesity and cancer. Front. Endocrinol. 9:396. 10.3389/fendo.2018.00396 30105001 PMC6077364

[B26] Deng X. Su R. Weng H. Huang H. Li Z. Chen J. (2018c). RNA N 6 -methyladenosine modification in cancers: current status and perspectives. Cell Res. 28, 507–517. 10.1038/s41422-018-0034-6 29686311 PMC5951805

[B27] Ding C. Zou Q. Ding J. Ling M. Wang W. Li H.. (2018). Increased N6-methyladenosine causes infertility is associated with FTO expression. J. Cell. Physiol. 233, 7055–7066. 10.1002/jcp.26507 29384212 PMC13482491

[B28] Dixit D. Xie Q. Rich J. N. Zhao J. C. (2017). Messenger RNA methylation regulates glioblastoma tumorigenesis. Cancer Cell 31, 474–475. 10.1016/j.ccell.2017.03.010 28399407 PMC6482444

[B29] Dominissini D. Moshitch-Moshkovitz S. Schwartz S. Salmon-Divon M. Ungar L. Osenberg S.. (2012). Topology of the human and mouse m6A RNA methylomes revealed by m6A-seq. Nature 485, 201–206. 10.1038/nature11112 22575960

[B30] Ensfelder T. T. Kurz M. Q. Iwan K. Geiger S. Matheisl S. Müller M.. (2018). ALKBH5-induced demethylation of mono- and dimethylated adenosine. Chem. Commun. 54, 8591–8593. 10.1039/C8CC03980A 30010678

[B31] Falnes P. Johansen R. F. Seeberg E. (2002). AlkB-mediated oxidative demethylation reverses DNA damage in *Escherichia coli*. Nature 419, 178–182. 10.1038/nature01048 12226668

[B32] Feng C. Liu Y. Wang G. Deng Z. Zhang Q. Wu W.. (2014). Crystal structures of the human RNA demethylase alkbh5 reveal basis for substrate recognition. J. Biol. Chem. 289, 11571–11583. 10.1074/jbc.M113.546168 24616105 PMC4002068

[B33] Flanagan J. U. Yosaatmadja Y. Teague R. M. Chai M. Z. L. Turnbull A. P. Squire C. J. (2012). Crystal structures of three classes of non-steroidal anti-inflammatory drugs in complex with aldo-keto reductase 1C3. PLoS ONE 7:e43965. 10.1371/journal.pone.0043965 22937138 PMC3429426

[B34] Forristal C. E. Wright K. L. Hanley N. A. Oreffo R. O. C. Houghton F. D. (2010). Hypoxia inducible factors regulate pluripotency and proliferation in human embryonic stem cells cultured at reduced oxygen tensions. Reproduction 139, 85–97. 10.1530/REP-09-0300 19755485 PMC2791494

[B35] Fu Y. Dai Q. Zhang W. Ren J. Pan T. He C. (2010). The AlkB domain of mammalian ABH8 catalyzes hydroxylation of 5-methoxycarbonylmethyluridine at the wobble position of tRNA. Angew. Chem. Int. Ed. 49, 8885–8888. 10.1002/anie.201001242 20583019 PMC3134247

[B36] Fu Y. Jia G. Pang X. Wang R. N. Wang X. Li C. J.. (2013). FTO-mediated formation of N6-hydroxymethyladenosine and N 6-formyladenosine in mammalian RNA. Nat. Commun. 4:2822. 10.1038/ncomms2822 23653210 PMC3658177

[B37] Gerken T. Girard C. A. Tung Y. C. L. Webby C. J. Saudek V. Hewitson K. S.. (2007). The obesity-associated FTO gene encodes a 2-oxoglutarate-dependent nucleic acid demethylase. Science 318, 1469–1472. 10.1126/science.1151710 17991826 PMC2668859

[B38] Gokhale N. S. McIntyre A. B. R. McFadden M. J. Roder A. E. Kennedy E. M. Gandara J. A.. (2016). N6-Methyladenosine in flaviviridae viral RNA genomes regulates infection. Cell Host Microbe 20, 654–665. 10.1016/j.chom.2016.09.015 27773535 PMC5123813

[B39] Gulati P. Cheung M. K. Antrobus R. Church C. D. Harding H. P. Tung Y. C. L.. (2013). Role for the obesity-related FTO gene in the cellular sensing of amino acids. Proc. Natl. Acad. Sci. U.S.A. 110, 2557–2562. 10.1073/pnas.1222796110 23359686 PMC3574930

[B40] Han Z. Niu T. Chang J. Lei X. Zhao M. Wang Q.. (2010). Crystal structure of the FTO protein reveals basis for its substrate specificity. Nature 464, 1205–1209. 10.1038/nature08921 20376003

[B41] Hao H. Hao S. Chen H. Chen Z. Zhang Y. Wang J.. (2019). N6-methyladenosine modification and METTL3 modulate enterovirus 71 replication. Nucleic Acids Res. 47, 362–374. 10.1093/nar/gky1007 30364964 PMC6326802

[B42] He Y. Hu H. Wang Y. Yuan H. Lu Z. Wu P.. (2018). ALKBH5 inhibits pancreatic cancer motility by decreasing long non-coding RNA KCNK15-AS1 methylation. Cell. Physiol. Biochem. 48, 838–846. 10.1159/000491915 30032148

[B43] Hesser C. R. Karijolich J. Dominissini D. He C. Glaunsinger B. A. (2018). N6-methyladenosine modification and the YTHDF2 reader protein play cell type specific roles in lytic viral gene expression during Kaposi's sarcoma-associated herpesvirus infection. PLoS Pathog. 14:e1006995. 10.1371/journal.ppat.1006995 29659627 PMC5919695

[B44] Hu B. B. Wang X. Y. Gu X. Y. Zou C. Gao Z. J. Zhang H.. (2019). N6-methyladenosine (m6A) RNA modification in gastrointestinal tract cancers: roles, mechanisms, and applications. Mol. Cancer 18:178. 10.1186/s12943-019-1099-7 31810483 PMC6898962

[B45] Huang H. Weng H. Chen J. (2020). m6A modification in coding and non-coding RNAs: roles and therapeutic implications in cancer. Cancer Cell 37, 270–288. 10.1016/j.ccell.2020.02.004 32183948 PMC7141420

[B46] Huang J. Yin P. (2018). Structural insights into N6-methyladenosine (m6A) modification in the transcriptome. Genomics Proteomics Bioinform. 16, 85–98. 10.1016/j.gpb.2018.03.001 29709557 PMC6112310

[B47] Huang Y. Su R. Sheng Y. Dong L. Dong Z. Xu H.. (2019). Small-molecule targeting of oncogenic FTO demethylase in acute myeloid leukemia. Cancer Cell 35, 677–691. 10.1016/j.ccell.2019.03.006 30991027 PMC6812656

[B48] Huang Y. Yan J. Li Q. Li J. Gong S. Zhou H.. (2015). Meclofenamic acid selectively inhibits FTO demethylation of m6A over ALKBH5. Nucleic Acids Res. 43, 373–384. 10.1093/nar/gku1276 25452335 PMC4288171

[B49] Iles M. M. Law M. H. Stacey S. N. Han J. Fang S. Pfeiffer R.. (2013). A variant in FTO shows association with melanoma risk not due to BMI. Nat. Genet. 45, 428–432. 10.1038/ng.2571 23455637 PMC3640814

[B50] Imam H. Khan M. Gokhale N. S. McIntyre A. B. R. Kim G. W. Jang J. Y. (2018). N6-methyladenosine modification of hepatitis b virus RNA differentially regulates the viral life cycle. Proc. Natl. Acad. Sci. U.S.A. 115, 8829–8834. 10.1073/pnas.1808319115 30104368 PMC6126736

[B51] Int C. Chen S. Zhou L. Wang Y. (2020). ALKBH5 - mediated - m 6 A demethylation of lncRNA PVT1 plays an oncogenic role in osteosarcoma. Cancer Cell Int. 20:34. 10.1186/s12935-020-1105-6 32021563 PMC6993345

[B52] Jia G. Fu Y. Zhao X. Dai Q. Zheng G. Yang Y.. (2011). N6-Methyladenosine in nuclear RNA is a major substrate of the obesity-associated FTO. Nat. Chem. Biol. 7, 885–887. 10.1038/nchembio.687 22002720 PMC3218240

[B53] Jia G. Yang C. G. Yang S. Jian X. Yi C. Zhou Z.. (2008). Oxidative demethylation of 3-methylthymine and 3-methyluracil in single-stranded DNA and RNA by mouse and human FTO. FEBS Lett. 582, 3313–3319. 10.1016/j.febslet.2008.08.019 18775698 PMC2577709

[B54] Jin H. Ying X. Que B. Wang X. Chao Y. Zhang H.. (2019). N6-methyladenosine modification of ITGA6 mRNA promotes the development and progression of bladder cancer. EBioMedicine 47, 195–207. 10.1016/j.ebiom.2019.07.068 31409574 PMC6796523

[B55] Kane S. E. Beemon K. (1985). Precise localization of m6A in rous sarcoma virus RNA reveals clustering of methylation sites: implications for RNA processing. Mol. Cell. Biol. 5, 2298–2306. 10.1128/MCB.5.9.2298 3016525 PMC366956

[B56] Kataoka H. Yamamoto Y. Sekiguchi M. (1983). A new gene (alkB) of *Escherichia coli* that controls sensitivity to methyl methane sulfonate. J. Bacteriol. 153, 1301–1307. 10.1128/JB.153.3.1301-1307.1983 6337994 PMC221777

[B57] Kawarada L. Suzuki T. Ohira T. Hirata S. Miyauchi K. Suzuki T. (2017). ALKBH1 is an RNA dioxygenase responsible for cytoplasmic and mitochondrial tRNA modifications. Nucleic Acids Res. 45, 7401–7415. 10.1093/nar/gkx354 28472312 PMC5499545

[B58] Kennedy E. M. Bogerd H. P. Kornepati A. V. R. Kang D. Ghoshal D. Marshall J. B.. (2016). Posttranscriptional m6A editing of HIV-1 mRNAs enhances viral gene expression. Cell Host Microbe 19, 675–685. 10.1016/j.chom.2016.04.002 27117054 PMC4867121

[B59] Kennedy E. M. Courtney D. G. Tsai K. Cullen B. R. (2017). Viral epitranscriptomics. J. Virol. 91, e02263–e02216. 10.1128/JVI.02263-16 28250115 PMC5391447

[B60] Krug R. M. Morgan M. A. Shatkin A. J. (1976). Influenza viral mRNA contains internal N6-methyladenosine and 5'-terminal 7-methylguanosine in cap structures. J. Virol. 20, 45–53. 10.1128/JVI.20.1.45-53.1976 1086370 PMC354964

[B61] Lang F. Singh R. K. Pei Y. Zhang S. Sun K. Robertson E. S. (2019). EBV epitranscriptome reprogramming by METTL14 is critical for viral-associated tumorigenesis. PLoS Pathog. 15:e1007796. 10.1371/journal.ppat.1007796 31226160 PMC6588254

[B62] Levanon E. Y. Eisenberg E. Yelin R. Nemzer S. Hallegger M. Shemesh R.. (2004). Systematic identification of abundant A-to-I editing sites in the human transcriptome. Nat. Biotechnol. 22, 1001–1005. 10.1038/nbt996 15258596

[B63] Li M. M. Nilsen A. Shi Y. Fusser M. Ding Y. H. Fu Y.. (2013). ALKBH4-dependent demethylation of actin regulates actomyosin dynamics. Nat. Commun. 4:2863. 10.1038/ncomms2863 23673617 PMC3674258

[B64] Li Q. Huang Y. Liu X. Gan J. Chen H. Yang C. G. (2016). Rhein inhibits AlkB repair enzymes and sensitizes cells to methylated DNA damage. J. Biol. Chem. 291, 11083–11093. 10.1074/jbc.M115.711895 27015802 PMC4900258

[B65] Li X. Xiong X. Zhang M. Wang K. Chen Y. Zhou J.. (2017). Base-resolution mapping reveals distinct m1A methylome in nuclear- and mitochondrial-encoded transcripts. Mol. Cell 68, 993–1005.e9. 10.1016/j.molcel.2017.10.019 29107537 PMC5722686

[B66] Li X. C. Jin F. Wang B. Y. Yin X. J. Hong W. Tian F. J. (2019). The m6A demethylase ALKBH5 controls trophoblast invasion at the maternal-fetal interface by regulating the stability of CYR61 mRNA. Theranostics 9, 3853–3865. 10.7150/thno.31868 31281518 PMC6587351

[B67] Li Z. Weng H. Su R. Weng X. Zuo Z. Li C.. (2017). FTO plays an oncogenic role in acute myeloid leukemia as a N6-methyladenosine RNA demethylase. Cancer Cell 31, 127–141. 10.1016/j.ccell.2016.11.017 28017614 PMC5234852

[B68] Lichinchi G. Gao S. Saletore Y. Gonzalez G. M. Bansal V. Wang Y.. (2016a). Dynamics of the human and viral m(6)A RNA methylomes during HIV-1 infection of T cells. Nat. Microbiol. 1:16011. 10.1038/nmicrobiol.2016.11 27572442 PMC6053355

[B69] Lichinchi G. Zhao B. S. Wu Y. Lu Z. Qin Y. He C.. (2016b). Dynamics of human and viral RNA methylation during zika virus infection. Cell Host Microbe 20, 666–673. 10.1016/j.chom.2016.10.002 27773536 PMC5155635

[B70] Linder B. Grozhik A. V. Olarerin-George A. O. Meydan C. Mason C. E. Jaffrey S. R. (2015). Single-nucleotide-resolution mapping of m6A and m6Am throughout the transcriptome. Nat. Methods 12, 767–772. 10.1038/nmeth.3453 26121403 PMC4487409

[B71] Liu F. Clark W. Luo G. Wang X. Fu Y. Wei J. (2016). ALKBH1-mediated tRNA demethylation regulates translation. Cell 167, 816–828.e16. 10.1016/j.cell.2016.09.038 27745969 PMC5119773

[B72] Liu Y. You Y. Lu Z. Yang J. Li P. Liu L.. (2019). N6-methyladenosine RNA modification–mediated cellular metabolism rewiring inhibits viral replication. Science 365, 1171–1176. 10.1126/science.aax4468 31439758

[B73] Malacrida A. Rivara M. Di Domizio A. Cislaghi G. Miloso M. Zuliani V.. (2020). 3D proteome-wide scale screening and activity evaluation of a new ALKBH5 inhibitor in U87 glioblastoma cell line. Bioorg. Med. Chem. 28:115300. 10.1016/j.bmc.2019.115300 31937477

[B74] Mauer J. Jaffrey S. R. (2018). FTO, m 6 A m, and the hypothesis of reversible epitranscriptomic mRNA modifications. FEBS Lett. 592, 2012–2022. 10.1002/1873-3468.13092 29754392

[B75] Mauer J. Luo X. Blanjoie A. Jiao X. Grozhik A. V. Patil D. P.. (2017). Reversible methylation of m6 Am in the 5′ cap controls mRNA stability. Nature 541, 371–375. 10.1038/nature21022 28002401 PMC5513158

[B76] McDonough M. A. Loenarz C. Chowdhury R. Clifton I. J. Schofield C. J. (2010). Structural studies on human 2-oxoglutarate dependent oxygenases. Curr. Opin. Struct. Biol. 20, 659–672. 10.1016/j.sbi.2010.08.006 20888218

[B77] Melstrom L. Chen J. (2020). RNA N 6 -methyladenosine modi fication in solid tumors : new therapeutic frontiers. Cancer Gene Ther. 27, 625–633. 10.1038/s41417-020-0160-4 31956264 PMC8627172

[B78] Molinie B. Wang J. Lim K. S. Hillebrand R. Lu Z. X. Van Wittenberghe N. (2016). M6 A-LAIC-seq reveals the census and complexity of the m6 A epitranscriptome. Nat. Methods 13, 692–698. 10.1038/nmeth.3898 27376769 PMC5704921

[B79] Monsen V. T. Sundheim O. Aas P. A. Westbye M. P. Sousa M. M. L. Slupphaug G. (2010). Divergent β-hairpins determine double-strand versus single-strand substrate recognition of human AlkB-homologues 2 and 3. Nucleic Acids Res. 38, 6447–6455. 10.1093/nar/gkq518 20525795 PMC2965238

[B80] Motorin Y. Lyko F. Helm M. (2009). 5-methylcytosine in RNA: detection, enzymatic formation and biological functions. Nucleic Acids Res. 38, 1415–1430. 10.1093/nar/gkp1117 20007150 PMC2836557

[B81] Müller T. A. Meek K. Hausinger R. P. (2010). Human AlkB homologue 1 (ABH1) exhibits DNA lyase activity at abasic sites. DNA Repair. 9, 58–65. 10.1016/j.dnarep.2009.10.011 19959401 PMC2818486

[B82] Narayan P. Ayers D. F. Rottman F. M. Maroney P. A. Nilsen T. W. (1987). Unequal distribution of N6-methyladenosine in influenza virus mRNAs. Mol. Cell. Biol. 7, 1572–1575. 10.1128/MCB.7.4.1572 3600638 PMC365250

[B83] Nettersheim D. Berger D. Jostes S. Kristiansen G. Lochnit G. Schorle H. (2019). N6-methyladenosine detected in RNA of testicular germ cell tumors is controlled by METTL3, ALKBH5, YTHDC1/F1/F2, and HNRNPC as writers, erasers, and readers. Andrology 7, 498–506. 10.1111/andr.12612 30903744

[B84] Niu Y. Lin Z. Wan A. Chen H. Liang H. Sun L.. (2019). RNA N6-methyladenosine demethylase FTO promotes breast tumor progression through inhibiting BNIP3. Mol. Cancer 18, 1–16. 10.1186/s12943-019-1004-4 30922314 PMC6437932

[B85] Padariya M. Kalathiya U. (2016). Structure-based design and evaluation of novel N-phenyl-1H-indol-2-amine derivatives for fat mass and obesity-associated (FTO) protein inhibition. Comput. Biol. Chem. 64, 414–425. 10.1016/j.compbiolchem.2016.09.008 27644082

[B86] PilŽys T. Marcinkowski M. Kukwa W. Garbicz D. Dylewska M. Ferenc K.. (2019). ALKBH overexpression in head and neck cancer: potential target for novel anticancer therapy. Sci. Rep. 9:13249. 10.1038/s41598-019-49550-x 31519943 PMC6744417

[B87] Pinello N. Sun S. Wong J. J. L. (2018). Aberrant expression of enzymes regulating m 6 A mRNA methylation: implication in cancer. Cancer Biol. Med. 15, 323–334. 10.20892/j.issn.2095-3941.2018.0365 30766746 PMC6372906

[B88] Rajecka V. Skalicky T. Vanacova S. (2019). The role of RNA adenosine demethylases in the control of gene expression. Biochim. Biophys. Acta Gene Regul. Mech. 1862, 343–355. 10.1016/j.bbagrm.2018.12.001 30550773

[B89] Roundtree I. A. Evans M. E. Pan T. He C. (2017). Dynamic RNA modifications in gene expression regulation. Cell 169, 1187–1200. 10.1016/j.cell.2017.05.045 28622506 PMC5657247

[B90] Safra M. Sas-Chen A. Nir R. Winkler R. Nachshon A. Bar-Yaacov D.. (2017). The m1A landscape on cytosolic and mitochondrial mRNA at single-base resolution. Nature 551, 251–255. 10.1038/nature24456 29072297

[B91] Sendinc E. Valle-garcia D. Dhall A. Gygi S. P. Sendinc E. Valle-garcia D.. (2019). PCIF1 catalyzes m6Am mRNA methylation to regulate gene expression. Mol. Cell 75, 620–630.e9. 10.1016/j.molcel.2019.05.030 31279659 PMC6688901

[B92] Shepard C. W. Simard E. P. Finelli L. Fiore A. E. Bell B. P. (2006). Hepatitis B virus infection: epidemiology and vaccination. Epidemiol. Rev. 28, 112–125. 10.1093/epirev/mxj009 16754644

[B93] Shriwas O. Priyadarshini M. Samal S. K. Rath R. Panda S. Das Majumdar S. K.. (2020). DDX3 modulates cisplatin resistance in OSCC through ALKBH5-mediated m6A-demethylation of FOXM1 and NANOG. Apoptosis 25, 233–246. 10.1007/s10495-020-01591-8 31974865

[B94] Singh B. Kinne H. E. Milligan R. D. Washburn L. J. Olsen M. Lucci A. (2016). Important role of FTO in the survival of rare panresistant triple-negative inflammatory breast cancer cells facing a severe metabolic challenge. PLoS ONE 11:e0159072. 10.1371/journal.pone.0159072 27390851 PMC4938613

[B95] Smemo S. Tena J. J. Kim K. H. Gamazon E. R. Sakabe N. J. Gómez-Marín C.. (2014). Obesity-associated variants within FTO form long-range functional connections with IRX3. Nature 507, 371–375. 10.1038/nature13138 24646999 PMC4113484

[B96] Song H. Feng X. Zhang H. Luo Y. Huang J. Lin M.. (2019). METTL3 and ALKBH5 oppositely regulate m6A modification of TFEB mRNA, which dictates the fate of hypoxia/reoxygenation-treated cardiomyocytes. Autophagy 15, 1419–1437. 10.1080/15548627.2019.1586246 30870073 PMC6613905

[B97] Su R. Dong L. Li C. Nachtergaele S. Wunderlich M. Qing Y.. (2018). R-2HG exhibits anti-tumor activity by targeting FTO/m6A/MYC/CEBPA signaling. Cell 172, 90–105.e23. 10.1016/j.cell.2017.11.031 29249359 PMC5766423

[B98] Sundheim O. Talstad V. A. Vågbø C. B. Slupphaug G. Krokan H. E. (2008). AlkB demethylases flip out in different ways. DNA Repair. 7, 1916–1923. 10.1016/j.dnarep.2008.07.015 18723127

[B99] Sundheim O. Vågbø C. B. Bjørås M. Sousa M. M. L. Talstad V. Aas P. A.. (2006). Human ABH3 structure and key residues for oxidative demethylation to reverse DNA/RNA damage. EMBO J. 25, 3389–3397. 10.1038/sj.emboj.7601219 16858410 PMC1523172

[B100] Svensen N. Jaffrey S. R. (2016). Fluorescent RNA aptamers as a tool to study RNA-modifying enzymes. Cell Chem. Biol. 23, 415–425. 10.1016/j.chembiol.2015.11.018 26877022 PMC4800036

[B101] Tan B. Gao S.-J. (2018). RNA epitranscriptomics: regulation of infection of RNA and DNA viruses by N6 -methyladenosine (m6A). Rev. Med. Virol. 28, 1–22. 10.1002/rmv.1983 29698584 PMC6339815

[B102] Tan B. Liu H. Zhang S. Zhang L. Cui X. Yuan H.. (2018). Viral and cellular N6 -methyladenosine (m6 A) and N6, 2′-O- dimethyladenosine (m6 Am) epitranscriptomes in KSHV life cycle. Nat. Microbiol. 3, 108–120. 10.1038/s41564-017-0056-8 29109479 PMC6138870

[B103] Tang B. Yang Y. Kang M. Wang Y. Wang Y. Bi Y.. (2020). M6A demethylase ALKBH5 inhibits pancreatic cancer tumorigenesis by decreasing WIF-1 RNA methylation and mediating Wnt signaling. Mol. Cancer 19, 1–15. 10.1186/s12943-019-1128-6 31906946 PMC6943907

[B104] Tang C. Klukovich R. Peng H. Wang Z. Yu T. Zhang Y.. (2017). ALKBH5-dependent m6A demethylation controls splicing and stability of long 3'-UTR mRNAs in male germ cells. Proc. Natl. Acad. Sci. U.S.A. 115, E325–E333. 10.1073/pnas.1717794115 29279410 PMC5777073

[B105] Thalhammer A. Bencokova Z. Poole R. Loenarz C. Adam J. O'Flaherty L.. (2011). Human AlkB homologue 5 is a nuclear 2-oxoglutarate dependent oxygenase and a direct target of hypoxia-inducible factor 1α (HIF-1α). PLoS ONE 6:e16210. 10.1371/journal.pone.0016210 21264265 PMC3021549

[B106] Tian L. F. Liu Y. P. Chen L. Tang Q. Wu W. Sun W.. (2020). Structural basis of nucleic acid recognition and 6mA demethylation by human ALKBH1. Cell Res. 30, 272–275. 10.1038/s41422-019-0233-9 32051559 PMC7054395

[B107] Tirumuru N. Zhao B. S. Lu W. Lu Z. He C. Wu L. (2016). N6-methyladenosine of HIV-1 RNA regulates viral infection and HIV-1 Gag protein expression. Microbiol. Infect. Dis. 5:e15528. 10.7554/eLife.15528.021 27371828 PMC4961459

[B108] Toh J. D. W. Sun L. Lau L. Z. M. Tan J. Low J. J. A. Tang C. W. Q.. (2015). A strategy based on nucleotide specificity leads to a subfamily-selective and cell-active inhibitor of N6-methyladenosine demethylase FTO. Chem. Sci. 6, 112–122. 10.1039/C4SC02554G 28553460 PMC5424463

[B109] Tsai K. Courtney D. G. Cullen B. R. (2018). Addition of m6A to SV40 late mRNAs enhances viral structural gene expression and replication. PLoS Pathog. 14:e1006919. 10.1371/journal.ppat.1006919 29447282 PMC5831754

[B110] Wang G. He Q. Feng C. Liu Y. Deng Z. Qi X.. (2014). The atomic resolution structure of human alkb homolog 7 (ALKBH7), a key protein for programmed necrosis and fat metabolism. J. Biol. Chem. 289, 27924–27936. 10.1074/jbc.M114.590505 25122757 PMC4183825

[B111] Wang R. Han Z. Liu B. Zhou B. Wang N. Jiang Q.. (2018). Identification of natural compound radicicol as a potent FTO inhibitor. Mol. Pharm. 15, 4092–4098. 10.1021/acs.molpharmaceut.8b00522 30063141

[B112] Wang T. Hong T. Huang Y. Su H. Wu F. Chen Y.. (2015). Fluorescein derivatives as bifunctional molecules for the simultaneous inhibiting and labeling of FTO protein. J. Am. Chem. Soc. 137, 13736–13739. 10.1021/jacs.5b06690 26457839

[B113] Wang Y. Li J. Han X. Wang N. Song C. Wang R.. (2019). Identification of clausine E as an inhibitor of fat mass and obesity-associated protein (FTO) demethylase activity. J. Mol. Recognit. 32:e2800. 10.1002/jmr.2800 31321808

[B114] Wei J. Liu F. Lu Z. Fei Q. Ai Y. He P. C.. (2018). Differential m 6 A, m 6 A m, and m 1 A demethylation mediated by FTO in the cell nucleus and cytoplasm. Mol. Cell 71, 973–985.e5. 10.1016/j.molcel.2018.08.011 30197295 PMC6151148

[B115] Weng H. Huang H. Chen J. (2019). RNA N 6-methyladenosine modification in normal and malignant hematopoiesis. Adv. Exp. Med. Biol. 1143, 75–93. 10.1007/978-981-13-7342-8_4 31338816

[B116] Westbye M. P. Feyzi E. Aas P. A. Vågbø C. B. Talstad V. A. Kavli B.. (2008). Human AlkB homolog 1 is a mitochondrial protein that demethylates 3-methylcytosine in DNA and RNA. J. Biol. Chem. 283, 25046–25056. 10.1074/jbc.M803776200 18603530 PMC3259822

[B117] Winkler R. Gillis E. Lasman L. Safra M. Geula S. Soyris C. (2019). m6A modification controls the innate immune response to infection by targeting type I interferons. Nat. Immunol. 20, 173–182. 10.1038/s41590-018-0275-z 30559377

[B118] Woo H. H. Chambers S. K. (2019). Human ALKBH3-induced m1A demethylation increases the CSF-1 mRNA stability in breast and ovarian cancer cells. Biochim. Biophys. Acta Gene Regul. Mech. 1862, 35–46. 10.1016/j.bbagrm.2018.10.008 30342176

[B119] Xu C. Liu K. Tempel W. Demetriades M. Aik W. Schofield C. J.. (2014). Structures of human ALKBH5 demethylase reveal a unique binding mode for specific single-stranded N6-methyladenosine RNA demethylation. J. Biol. Chem. 289, 17299–17311. 10.1074/jbc.M114.550350 24778178 PMC4067165

[B120] Xu D. Shao W. Jiang Y. Wang X. Liu Y. Liu X. (2017). FTO expression is associated with the occurrence of gastric cancer and prognosis. Oncol. Rep. 38, 2285–2292. 10.3892/or.2017.5904 28849183

[B121] Xue M. Zhao B. S. Zhang Z. Lu M. Harder O. Chen P.. (2019). Viral N 6-methyladenosine upregulates replication and pathogenesis of human respiratory syncytial virus. Nat. Commun. 10:4595. 10.1038/s41467-019-12504-y 31597913 PMC6785563

[B122] Yang C. G. Yi C. Duguid E. M. Sullivan C. T. Jian X. Rice P. A.. (2008). Crystal structures of DNA/RNA repair enzymes AlkB and ABH2 bound to dsDNA. Nature 452, 961–965. 10.1038/nature06889 18432238 PMC2587245

[B123] Yang P. Wang Q. Liu A. Zhu J. Feng J. (2019). ALKBH5 holds prognostic values and inhibits the metastasis of colon cancer. Pathol. Oncol. Res. 26, 1615–1623. 10.1007/s12253-019-00737-7 31506804

[B124] Yang S. Wei J. Cui Y. H. Park G. Shah P. Deng Y.. (2019). m6A mRNA demethylase FTO regulates melanoma tumorigenicity and response to anti-PD-1 blockade. Nat. Commun. 10:2782. 10.1038/s41467-019-10669-0 31239444 PMC6592937

[B125] Ye F. Chen E. R. Nilsen T. W. (2017). Kaposi's sarcoma-associated herpesvirus utilizes and manipulates RNA N6- adenosine methylation to promote lytic replication. J. Virol. 91, 1–21. 10.1128/JVI.00466-17 28592530 PMC5533915

[B126] Ye F. Lei X. Gao S. J. (2011). Mechanisms of kaposi's sarcoma-associated herpesvirus latency and reactivation. Adv. Virol. 2011:193860. 10.1155/2011/193860 21625290 PMC3103228

[B127] Yu J. Shen L. Liu Y. Ming H. Zhu X. Chu M.. (2020). The m6A methyltransferase METTL3 cooperates with demethylase ALKBH5 to regulate osteogenic differentiation through NF-κB signaling. Mol. Cell. Biochem. 463, 203–210. 10.1007/s11010-019-03641-5 31643040

[B128] Zhang C. Samanta D. Lu H. Bullen J. W. Zhang H. Chen I.. (2016a). Hypoxia induces the breast cancer stem cell phenotype by HIF-dependent and ALKBH5-mediated m6A-demethylation of NANOG mRNA. Proc. Natl. Acad. Sci. U.S.A. 113, E2047–E2056. 10.1073/pnas.1602883113 27001847 PMC4833258

[B129] Zhang C. Zhang M. Ge S. Huang W. Lin X. Gao J.. (2019). Reduced m6A modification predicts malignant phenotypes and augmented Wnt/PI3K-Akt signaling in gastric cancer. Cancer Med. 8, 4766–4781. 10.1002/cam4.2360 31243897 PMC6712480

[B130] Zhang C. Zhi W. I. Lu H. Samanta D. Chen I. Gabrielson E.. (2016b). Hypoxia-inducible factors regulate pluripotency factor expression by ZNF217-and ALKBH5-mediated modulation of RNA methylation in breast cancer cells. Oncotarget 7, 64527–64542. 10.18632/oncotarget.11743 27590511 PMC5323097

[B131] Zhang J. Guo S. Piao H. Y. Wang Y. Wu Y. Meng X.. (2019). ALKBH5 promotes invasion and metastasis of gastric cancer by decreasing methylation of the lncRNA NEAT1. J. Physiol. Biochem. 75, 379–389. 10.1007/s13105-019-00690-8 31290116 PMC6728298

[B132] Zhang M. Yang S. Nelakanti R. Zhao W. Liu G. Li Z.. (2020). Mammalian ALKBH1 serves as an N 6-mA demethylase of unpairing DNA. Cell Res. 30, 197–210. 10.1038/s41422-019-0237-5 32051560 PMC7054317

[B133] Zhang S. Zhao B. S. Zhou A. Lin K. Zheng S. Lu Z.. (2017). m6A demethylase ALKBH5 maintains tumorigenicity of glioblastoma stem-like cells by sustaining FOXM1 expression and cell proliferation program. Cancer Cell 31, 591–606.e6. 10.1016/j.ccell.2017.02.013 28344040 PMC5427719

[B134] Zhang X. Wei L. H. Wang Y. Xiao Y. Liu J. Zhang W.. (2019). Structural insights into FTO's catalytic mechanism for the demethylation of multiple RNA substrates. Proc. Natl. Acad. Sci. U.S.A. 116, 2919–2924. 10.1073/pnas.1820574116 30718435 PMC6386707

[B135] Zhao W. Qi X. Liu L. Liu Z. Ma S. Wu J. (2020). Epigenetic regulation of m6A modifications in human cancer. Mol. Ther. Nucleic Acids 19, 405–412. 10.1016/j.omtn.2019.11.022 31887551 PMC6938965

[B136] Zhao X. Yang Y. Sun B. F. Shi Y. Yang X. Xiao W.. (2014a). FTO-dependent demethylation of N6-methyladenosine regulates mRNA splicing and is required for adipogenesis. Cell Res. 24, 1403–1419. 10.1038/cr.2014.151 25412662 PMC4260349

[B137] Zhao X. Yang Y. Sun B. F. Zhao Y. L. Yang Y. G. (2014b). FTO and obesity: mechanisms of association. Curr. Diab. Rep. 14:486. 10.1007/s11892-014-0486-0 24627050

[B138] Zheng G. Cox T. Tribbey L. Wang G. Z. Iacoban P. Booher M. E.. (2014). Synthesis of a FTO inhibitor with anticonvulsant activity. ACS Chem. Neurosci. 5, 658–665. 10.1021/cn500042t 24834807 PMC4140589

[B139] Zheng G. Dahl J. A. Niu Y. Fedorcsak P. Huang C. M. Li C. J.. (2013). ALKBH5 is a mammalian RNA demethylase that impacts RNA metabolism and mouse fertility. Mol. Cell 49, 18–29. 10.1016/j.molcel.2012.10.015 23177736 PMC3646334

[B140] Zheng Q. Hou J. Zhou Y. Li Z. Cao X. (2017). The RNA helicase DDX46 inhibits innate immunity by entrapping m 6 A-demethylated antiviral transcripts in the nucleus. Nat. Immunol. 18, 1094–1103. 10.1038/ni.3830 28846086

[B141] Zhou Y. Hambly B. D. McLachlan C. S. (2017). FTO associations with obesity and telomere length. J. Biomed. Sci. 24, 1–7. 10.1186/s12929-017-0372-6 28859657 PMC5580219

[B142] Zhu H. Gan X. Jiang X. Diao S. Wu H. Hu J. (2019). ALKBH5 inhibited autophagy of epithelial ovarian cancer through miR-7 and BCL-2. J. Exp. Clin. Cancer Res. 38, 1–15. 10.1186/s13046-019-1159-2 30987661 PMC6463658

[B143] Zhu Z. Qian Q. Zhao X. Ma L. Chen P. (2020). N6-methyladenosine ALKBH5 promotes non-small cell lung cancer progress by regulating TIMP3 stability. Gene 731:144348. 10.1016/j.gene.2020.144348 31927006

[B144] Zou S. Toh J. D. W. Wong K. H. Q. Gao Y. G. Hong W. Woon E. C. Y. (2016). N 6 -Methyladenosine: a conformational marker that regulates the substrate specificity of human demethylases FTO and ALKBH5. Sci. Rep. 6:25677. 10.1038/srep25677 27156733 PMC4860565

